# Combinatorial Modeling of Chromatin Features Quantitatively Predicts DNA Replication Timing in *Drosophila*

**DOI:** 10.1371/journal.pcbi.1003419

**Published:** 2014-01-23

**Authors:** Federico Comoglio, Renato Paro

**Affiliations:** 1 Department of Biosystems Science and Engineering, ETH Zurich, Basel, Switzerland; 2 Faculty of Science, University of Basel, Basel, Switzerland; Harvard University, United States of America

## Abstract

In metazoans, each cell type follows a characteristic, spatio-temporally regulated DNA replication program. Histone modifications (HMs) and chromatin binding proteins (CBPs) are fundamental for a faithful progression and completion of this process. However, no individual HM is strictly indispensable for origin function, suggesting that HMs may act combinatorially in analogy to the histone code hypothesis for transcriptional regulation. In contrast to gene expression however, the relationship between combinations of chromatin features and DNA replication timing has not yet been demonstrated. Here, by exploiting a comprehensive data collection consisting of 95 CBPs and HMs we investigated their combinatorial potential for the prediction of DNA replication timing in *Drosophila* using quantitative statistical models. We found that while combinations of CBPs exhibit moderate predictive power for replication timing, pairwise interactions between HMs lead to accurate predictions genome-wide that can be locally further improved by CBPs. Independent feature importance and model analyses led us to derive a simplified, biologically interpretable model of the relationship between chromatin landscape and replication timing reaching 80% of the full model accuracy using six model terms. Finally, we show that pairwise combinations of HMs are able to predict differential DNA replication timing across different cell types. All in all, our work provides support to the existence of combinatorial HM patterns for DNA replication and reveal cell-type independent key elements thereof, whose experimental investigation might contribute to elucidate the regulatory mode of this fundamental cellular process.

## Author Summary

Before a cell divides, its genome must be faithfully duplicated to ensure that the daughter cell receives an exact copy of the parental genetic material. However, this process requires disruption of chromatin, the combination of DNA and histone proteins, whose structure and function have to be readily restored afterwards. This is achieved through a nuclear process known as DNA replication, which represents the basis for biological inheritance. In eukaryotes, genome replication starts from distinct genomic locations termed replication origins. Origins fire in a temporally regulated, cell-type dependent manner and timing of DNA replication is therefore the result of this concerted origin activation. However, replication timing is not encoded in the genome and its regulatory mode remains to a large degree unresolved. Here, we systematically study the relationship between chromatin, represented by histone modifications and chromatin binding proteins, and DNA replication timing. We report combinatorial histone modification patterns exhibiting regulatory potential for this process and we characterize those elements that might contribute to further elucidate the regulatory mode of this fundamental cellular process.

## Introduction

In eukaryotes, DNA replication is regulated both in time and space and initiates at multiple origins along the genome [1]. When averaged over a cell population, each genomic region shows reproducible replication timing in S-phase [2], [3]. The timing of replication is a mitotically stable cell-type specific feature of chromosomes [4] that was recently legitimated as an epigenetic feature [5]. For example, many tissue specific genes that are subject to developmental regulation are early replicating in their tissue of expression but rather late replicating in other tissues. Conversely, housekeeping genes expressed in almost all tissues are replicated in the first half of the S-phase [6], [7].

From an epigenetic point of view DNA replication constitutes a periodic window of both risk and opportunity. On one hand, established chromatin patterns of genome regulation are challenged by their disruption at the time of replication [8]. On the other hand, the same process paves the way for epigenetic changes and hence adaptation of cells to new cues. Our current understanding of the molecular mechanisms underlying eukaryotic DNA replication is the result of decades of experimental work that exploited model organisms as diverse as budding yeast, *Xenopus laevis* and *Drosophila melanogaster* [9]. Very recent work shed light on basic principles that regulate DNA replication timing at a global level [10]–[13]. Genome-wide profiling of DNA replication timing substantially contributed to these findings and a number of replication timing profiles are now available for different organisms and cell lines [4], [14]–[16]. The concurrent release of genome-wide profiles of histone modifications (HMs) and chromatin binding proteins (CBPs) through large scale genomic projects such as modENCODE and ENCODE represents a timely opportunity to systematically investigate the connection between replication timing and chromatin landscape. To date, chromatin feature levels have been individually correlated genome-wide to replication timing in different organisms [4], [15], [17], [18] and this studied extended single-locus-based observations to a genome-wide scale. Particularly, it is now accepted that euchromatin, gene dense, transcriptionally active regions of the genome preferentially replicate in early S-phase, as opposed to constitutive heterochromatin, repetitive, transcriptionally inactive regions that remain condensed throughout the cell cycle [1]. However, the observation that gene expression requires to be averaged over chromatin domains to strongly correlate with their replication timing [2], [19], suggested that this domain-like organization of replication timing might be regulated through higher-order chromatin structure [17], [20]. This, in turn, contributed to the development of qualitative models in which the chromosome accessibility of a domain affects its replication timing [2], [20].

Recent work linked HMs and CBPs levels to gene expression by means of quantitative statistical models [21]–[25], singling out a small number of HMs predicting the transcriptional output with high accuracy. However, HMs and CBPs also play a pivotal role in ensuring faithful completion of the DNA replication program [26]–[30]. As no individual HM has been found to be essential for origin function to date, it is likely that HMs act combinatorially in regulating DNA replication timing. Indeed, the view of chromatin as a platform for the assembly of different protein complexes in conjunction with the combinatorial nature of HMs led to the formulation of the hotly debated histone code hypothesis, in which specific combinations of HMs determine unique biological outputs [31]–[34]. Although proposed as a regulatory mechanism of chromatin-templated processes and well investigated for transcriptional regulation, this concept has to our knowledge not yet been demonstrated for DNA replication. Seminal work by Eaton *et al.* [15] tightened the link between chromatin features and DNA replication timing by showing that clusters of chromatin features are predictive for early origin activity and changes thereof in *Drosophila*. Here, we set out to systematically characterize this link and investigate the combinatorial relevance of chromatin features in predicting replication timing. Using a comprehensive data collection encompassing 95 HMs and CBPs profiled by the modENCODE project or independent studies in *Drosophila* cell lines, we asked the following five questions: i) Is there a quantitative relationship between HMs and CBPs levels and DNA replication timing? ii) Do these features act combinatorially and if yes, do HMs and CBPs convey redundant or distinct information? iii) Which features contribute the most in this relationship? iv) Do these rules apply genome-wide? v) Can these rules be generalized to various cell types? We addressed these points using Lasso (Least Absolute Shrinkage and Selection Operator), an *L*^1^-norm regularized linear model [35]. We systematically analyzed the predictive power of different subsets of chromatin features and combinatorial schemes thereof, applied feature importance analyses to obtain a simplified, biologically interpretable model and revealed cell-type independent combinations of chromatin features potentially impacting origin firing and likely to be conserved across species.

## Results/Discussion

### Individual chromatin features exhibit limited predictive power on DNA replication timing

Recent studies reported moderate correlations between single chromatin features and DNA replication timing [15], [18], [36], [37]. However, these analyses were based on a rather limited number of genome-wide profiles. Here, we considered a genome-wide replication timing profile generated by [15] using tiling arrays and investigated the individual predictive power of a comprehensive set of 95 chromatin features (30 HMs - more precisely 28 HMs and 2 histone variants hereinafter collectively referred to as HMs - and 65 CBPs) profiled in *Drosophila* S2 cells using ChIP-chip or ChIP-Seq and generated by modENCODE [38] or independent studies. The goal of our study is to predict the replication timing across the *Drosophila* S2 genome. To this purpose, as the precise genomic coordinates of replication origins remain rather elusive in metazoans, we first considered a set of 7552 unique promoters (see Methods) for model learning. Several studies reported that replication initiation sites are associated with transcriptional units [14], [36], [39] and share common sequence motifs thereof [36]. In addition, the majority of ORC binding sites overlap with transcription start sites (TSSs) in *Drosophila* [40]. Feature levels and replication timing were therefore estimated for each promoter in a 1 kb window centered on its TSS (see Methods). As we integrated data sets generated by different laboratories and platforms, we first hierarchically clustered chromatin feature profiles at promoters and verified that feature levels reflected known biological associations between CBPs and HMs (Supplementary Figure S1). Then, for each feature we fitted cross-validated univariate linear regression models to analyze its predictive power on promoter-proximal replication timing. Our results confirm that individual features are rather poor predictors of replication timing (Supplementary Figure S2). Single HMs are on average significantly more predictive than individual CBPs (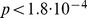, two-sided t-test), but only few of them, i.e. H4K8ac, H3K36me1, H3K18ac, H4K5ac, H3K4me1, can predict replication timing with an accuracy (Pearson's correlation coefficient, hereinafter PCC or *ρ*) of 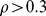. As previously shown [15], histone variants H2Av and H3.3 are positively correlated with replication timing. In addition, we found that levels of H4K5ac are predictive for early replication and that levels of H4K20me1, total H4 and linker histone H1, are individually predictive for late replication (Supplementary Figure S2). These results support the current view in which levels of acetylated and mono-methylated histones, localizing within euchromatin and marking accessible chromatin, are predictive for early replication, in contrast to levels of heterochromatic marks. Among CBPs, RNA Pol II (Pol II) and chromatin remodelers (such as ISWI, NURF and GAF) were previously shown to correlate with early replication timing in *Drosophila* [15]. Besides confirming these observations, our analysis highlights two CBPs, i.e. the chaperone protein Hsp90 and the ATP-dependent chromatin-remodeling factor dMi2, as top-ranked features predictive for early replication. The latter is involved in rapid nucleosome turnover, a distinguishing feature of origins of replication and promoters [41], and has been very recently implicated in regulation of higher-order chromatin structures and local decondensation of chromatin in *Drosophila* [42]. Hsp90 is involved in a number of chromatin processes [43]. Particularly, chromatin-associated Hsp90 is widespread genome-wide, where it binds to the TSSs of Pol II paused genes [44]. Our finding suggests that Hsp90 might be involved in regulating the timing of replication origin firing via a transcriptional-dependent or independent mechanism. However, experimental work will be required to detail this mechanism and to exclude an indirect role of Hsp90 as a marker of accessible chromatin. All in all, the limited predictive power of single features led us to hypothesize the existence of a combinatorial interplay between chromatin features enabling an accurate description of their relationship with replication timing. In the next sections, we test this hypothesis.

### Combinatorial contribution of chromatin binding proteins to replication timing prediction

Quantitative modeling of the relationship between chromatin features and DNA replication timing requires testing of combinatorial patterns of chromatin features. In this high dimensional space, over-fitting represents a significant risk and therefore model regularization and cross-validation are required to effectively minimize it. Thus, our analysis is based on the statistical model Lasso (Least Absolute Shrinkage and Selection Operator, see Methods for details) [35], [45], a regularized linear model that penalizes model complexity through an *L*^1^ norm penalty. As a consequence, Lasso coefficients are sparse and feature selection is performed implicitly, facilitating model interpretation [35], [46]. Regularized regression methods have been previously employed to discover transcription factor binding motifs [47] and Lasso was very recently applied to predict RNA expression and promoter-proximal pausing from CBPs profiles [48].

**Figure 1:**
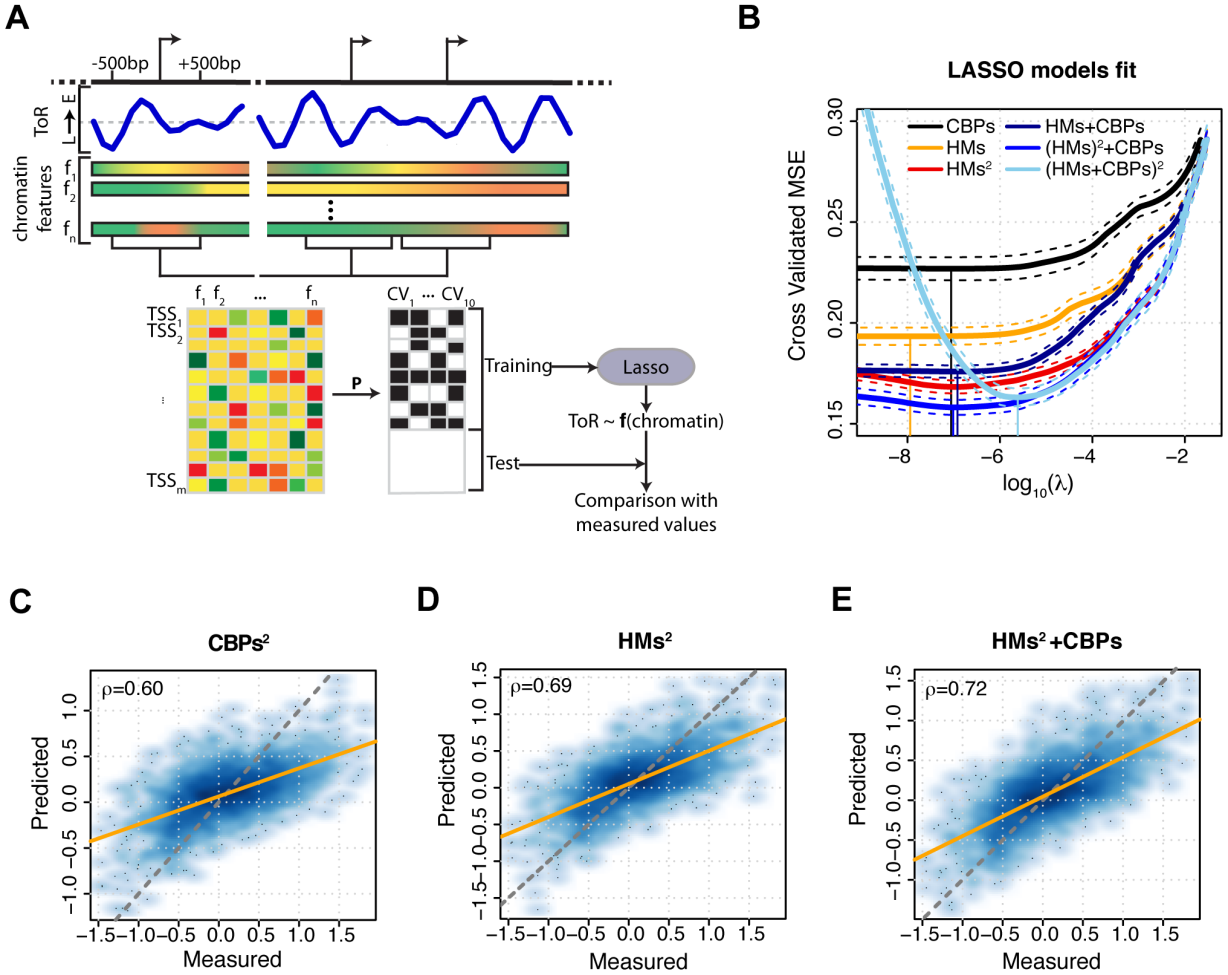
Schematic representation of the modeling framework and combinatorial predictive power of chromatin features. (A) Schematic illustration of the modeling framework. DNA replication timing (ToR, blue line) and chromatin feature signals (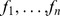 indicated by gradient filled rectangles) were quantified for each promoter in a 1 kb window centered on its TSS (). The resulting input data matrix is shown (bottom left), where feature levels are encoded by different colors ranging from dark green to red. TSSs were then randomized according to a permutation *P* and split in training and test sets. The training set is used to train a Lasso model using 10-fold cross validation. At each model fit (CV_1_ to CV_10_), a TSS can either be assigned to the training set (black square) or to the test set (white square). The model was then used to infer the replication timing of promoters in the test set and the model accuracy is evaluated with respect to their experimentally measured replication timing. (B) Cross-validated mean squared error (CV-MSE) as a function of the regularization parameter (log_10_(*λ*)) for different Lasso models trained with ten fold cross-validation. The average CV-MSE is reported as solid line, with minimum and maximum CV-MSE drawn as dashed lines. A vertical line reaching a CV-MSE curve indicates the value of *λ* that was used to generate predictions from the corresponding model. The different sets of features used for model training are indicated in the legend. (C–E) Predicted versus experimentally measured replication timing of the test set represented as smoothed color density scatter plot. Model predictions were generated using second-order interactions between CBPs (CBPs^2^, C), HMs (HMs^2^, D) and HMs^2^+CBPs (E). Prediction accuracies are Pearson correlation coefficients. Orange lines indicate the model fit, whereas dashed gray lines indicate the bisector 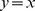.

Figure 1A illustrates our modeling framework. First, unique promoters were randomly partitioned into training and test sets (see Methods). Lasso models were then trained with ten-fold cross validation on the training set. To this purpose, the training set was randomly partitioned into ten subsets of equal size. Then, at each round of cross validation one subset was used in turn as validation set, while the model was learnt on the remaining nine subsets. The resulting ten models were averaged to obtain the cross-validated model. Prediction accuracy was evaluated on the test set and defined as the PCC between measured and predicted replication timing.

We started by analyzing the combinatorial predictive power of CBPs. When CBP levels were jointly considered, the model achieved an accuracy of 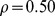 (Figure 1B and Supplementary Figure S3A, B). Although significantly higher than the predictive power of any individual protein, this value is still modest. Thus, we investigated whether the addition of multiplicative interaction terms, in the form of second-order interactions, could raise the predictive power of CBPs. We found that allowing for pairwise interactions between CBPs significantly improved the model accuracy (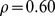, Figure 1C and Supplementary Figure S3A), suggesting that CBPs might combinatorially contribute to the regulation of replication timing. Notably, the higher predictive power of the latter model is not a mere consequence of an increased complexity as consideration of third-order interactions led to predictions that did not correlate any better with measured replication timing (*ρ* = 0.61, Supplementary Figure S3A, C). Taken together, these results indicate that CBPs and their pairwise interactions can account for a moderate yet substantial fraction, approximately 35%, of the variation in replication timing.

### Combinatorial contribution of histone modifications to replication timing prediction

We next analyzed the relationship between HM levels and DNA replication timing. As for CBPs, we combined HMs using Lasso. The prediction accuracy achieved with HMs (*ρ* = 0.61, Figure 1B and Supplementary Figure S3A,D) is significantly higher than what we previously obtained with CBPs and as for the latter, significantly higher than the predictive power of any individual feature. As the histone code hypothesis postulated that HMs act combinatorially in regulating chromatin processes, with a one-to-one mapping between HM combinations and biological outcomes [31]–[33], we tested whether considering multiplicative second-order interactions between HMs could further increase the accuracy of the previous model. Inclusion of these combinations significantly raised the model accuracy from *ρ* = 0.61 to *ρ* = 0.69 (Figure 1B,D), suggesting that a combinatorial interplay between pairs of HMs might modulate DNA replication timing in *Drosophila*. This result suggested us to test whether more complex combinations, in the form of multiplicative third-order interactions between HMs, could bear even more predictive power than pairwise interactions. On the same line as for CBPs, we found that the prediction accuracy did not significantly increase (*ρ* = 0.69, Supplementary Figure S3A,E) solely as a consequence of a higher model complexity. Although this result implies that combinatorial patterns of HMs exhibit low complexity, in line with observations *in vivo* pertaining gene expression regulation [34], a very recent computational analysis showed that a simple histone code, based on modification at two histone residues, may suffice to generate a number of different circuits featuring heritable bistability [49].

In summary, we showed that HMs and their pairwise interactions are more predictive for replication timing than the corresponding terms involving CBPs. This result suggested to analyze the joint predictive power of CBPs and HMs and to test their redundancy for replication timing predictions.

### Combinations of histone modifications and chromatin binding proteins predict replication timing with high accuracy

To test whether CBPs and HMs convey redundant information on replication timing, we trained a Lasso model by jointly considering these two sets of features. We found that predictions based on combinations of CBPs and HMs exhibit a significantly lower cross-validated mean squared error (MSE) than the models trained on CBPs or HMs alone (Figure 1B) and thereby outperformed (*ρ* = 0.67, Supplementary Figure S4A) the accuracy of models solely based on CBPs (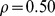, Supplementary Figure S3B) or HMs (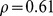, Supplementary Figure S3D). However, this result indicates a partial redundancy between CBPs and HMs, which was further supported by a simple analysis of model residuals. As residuals are differences between measured and estimated DNA replication timing, they can be seen as information about replication timing that can not be explained by the model. Thus, we first considered the residuals of the model trained on CBP levels. Then, we tested whether HMs exhibit any predictive power for these residuals. Under the hypothesis that CBPs and HMs convey redundant information on replication timing, no correlation between model predictions and residuals is expected. Conversely, we found that HM levels can predict replication timing residuals with a highly significant yet moderate accuracy (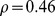). A similar result, despite a lower predictive power (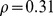), was obtained when CBPs were used to predict the residuals of the model trained solely on HMs. These results suggested us to investigate whether the introduction of CBPs could comparably raise the predictive power of second-order interactions between HMs. Indeed, CBPs in conjunction with pairwise interactions of HMs led to a model able to predict replication timing with higher accuracy (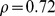, Figure 1E) than HMs alone (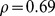, Figure 1D) and significantly reduced the cross-validated MSE as compared to the latter (Figure 1B). Finally, we tested whether allowing multiplicative cross-interactions between HMs and CBPs could further increase our ability to predict replication timing. However, despite a large increase in complexity this model did not outperform the previous one simply based on CBPs and interactions between HMs (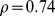, Supplementary Figure S4B), confirming once again that in our framework prediction accuracies are not a sheer consequence of the number of features. Similarly, further extension of the model by inclusion of RNA-Seq-based gene expression levels from [50] or multivariate Hidden Markov Model-based chromatin states [51], [52] from modENCODE [53] did not significantly improve prediction accuracy (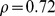 and data not shown). Taken together, these results indicate that CBPs and HMs are able to explain slightly more than 50% of the variation in DNA replication timing and suggest that these two sets of features contain partially complementary information that, when jointly captured, enable accurate predictions.

### Thorough model analysis reveals combinations of histone modifications harboring most of the information about DNA replication timing

**Figure 2:**
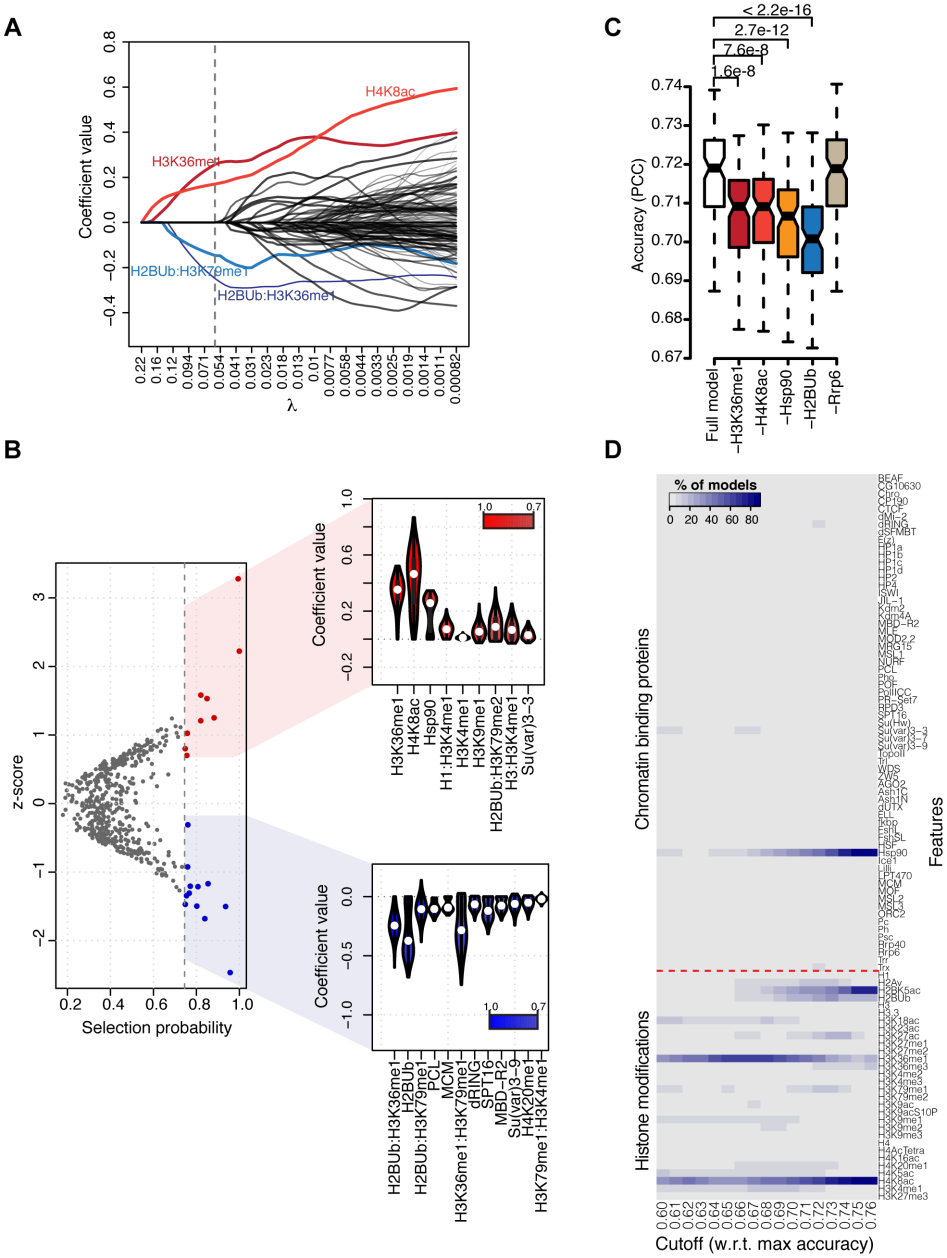
Feature importance analysis and simplified models. (A) Values of the model coefficients along the *λ*-path, i.e. the sequence of values of the regularization parameter *λ* used to fit the model. The *λ*-path is truncated at the value of *λ* used for model predictions. Line thickness is proportional to the total number of models in which a non-zero coefficient is assigned to the corresponding feature. The vertical dashed line denotes the value of *λ* yielding the selected simplified model solely based on the four indicated terms. (B) Scatter plot of model features according to their *z*-scores and bootstrap-Lasso selection probabilities (*p*). Features with 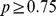 are colored in red (positive coefficient values) or blue (negative coefficient values) and their coefficient distributions are shown on the right as violin plots. Features are ranked by decreasing selection probabilities. (C) Boxplot of prediction accuracies (PCC on test sets) of 100 Lasso models where the indicated feature was excluded from the model fit. Rrp6 was used as control, as stability analysis indicated no significant role for this feature in predicting replication timing. *p*-values were obtained using a two-sided Wilcoxon rank sum test. (D) Frequency of appearance of chromatin features in four-features simplified models as a function of their model accuracy with respect to the full model. Only simplified models reaching at least 60% of the full model accuracy are shown.

Here, we consider the Lasso model based on CBPs and pairwise interactions between HMs, we analyze feature importance and identify simplified models able to achieve a substantial fraction of the full model accuracy using few chromatin features. Although a measure of feature importance is not directly available for Lasso, different approaches can be employed to overcome this issue. First, the geometric constraints imposed to Lasso solutions result in an implicit feature selection [45]. This process depends on the extent of the regularization applied to the model, tuned by the parameter *λ*. The stronger the regularization, i.e. the higher *λ*, the smaller the number of selected features (see Methods for details). Consequently, there exists an entire set of Lasso models along the *λ*-path (i.e. the sequence of values of *λ* used to fit the model) each characterized by different model coefficients. Figure 2A shows the model coefficient curves along this path. Searching for simplified models is equivalent to identify those models with few non-zero coefficients and relatively high accuracy along the *λ*-path. Therefore, we considered all models reaching at least 70% of the full model accuracy and identified a first simplified model solely based on four terms involving four histone modifications, i.e. H3K36me1, H4K8ac, H2BUb and H3K79me1, able to reach an accuracy of 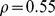, namely 76% of the full model accuracy. H3K36me1 and H4K8ac are predictive for early replication whereas pairwise interactions between H2BUb and H3K36me1 or H3K79me1 are predictive for late replication (Figure 2A). Interestingly, H3K36me1 exhibits opposite effects depending on whether it is considered alone or through its interaction with H2BUb, suggesting a context-dependent role of this modification.

If a group of features is characterized by high pairwise correlations, Lasso tends to arbitrarily select only one representative feature from the group [54]. However when not a single, but several models are fit on resampled data, feature selection frequencies can be used to estimate variable importance. Features indispensable to achieve high prediction accuracy will be selected with high frequency whereas redundant features or representatives of group effects will dilute their selection probabilities. Hence, to test whether the HMs identified above are indispensable for accurate predictions or rather representatives of functional groups of HMs, we estimated feature selection probabilities using bootstrap-Lasso [48]. In this method, data points are repeatedly sampled with replacement (bootstrap) to generate data sets used to train a full set of Lasso models along a fixed *λ*-path. The selection probability of each feature can then be estimated by considering the normalized frequency of non-zero coefficients (see Methods). Our bootstrap-Lasso analysis indicates that H3K36me1 and H4K8ac, followed by Hsp90, are selected with high probability and predictive for early replication timing (Figure 2B). Conversely, three terms involving H2BUb, namely the modification alone and its interaction with H3K36me1 and H3K79me1, are characterized by high selection probabilities and predictive for late replication. These results indicate that the previously identified simplified model was based on indispensable features and led us to test whether the addition of Hsp90 and H2BUb could further raise its predictive power. Indeed, we found that the inclusion of these two features substantially raised the prediction accuracy of the simplified model to 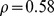, thus reaching 80% of the full model accuracy. The overall significance of H3K36me1, H4K8ac, H2BUb and Hsp90 in predicting replication timing was further substantiated using a bootstrap-based approach in which these features were individually excluded from the model fit (see Methods, Figure 2C). Furthermore, since the Hsp90 profile was generated by ChIP-Seq, we technically excluded that this feature was selected solely based on sequencing depth as Hsp90 was neither among highest coverage features (Supplementary Figure S5A) nor a strong correlation between coverage and individual predictive power of ChIP-Seq-derived chromatin features emerged in our analysis (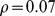, Supplementary Figure S5B).

Finally, we independently sought for simplified models using exhaustive model search as proposed in [21], [22]. To this purpose, we considered all possible combinations of two, three and four chromatin features and used each combination to train a multivariate linear regression model (see Methods). Prediction accuracies were recorded for a total of 3 188 010 models (Supplementary Figure S6). The Bayesian Information Criterion (BIC) was used to account for model complexity and monotonically decreased as more features were introduced, indicating that including up to four features is beneficial for prediction accuracy (Supplementary Figure S6A) irrespective of model complexity. Notice that we could not generate models with five or more features as the number of *k*-features models (*n*) grows with the binomial coefficient 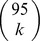, i.e. 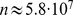 for 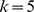. However, we determined top two-features (H3K36me1, H4K8ac, 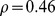), top-three features (Hsp90, H2BK5ac, H4K8ac, 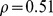) and top-four features (H2BUb, H3K36me1, H3K36me3, Hsp90, 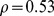) models. Although these combinations differ slightly from the ones determined via bootstrap-Lasso, all features therein belong to at least one top-ranked simplified model. Moreover, by analyzing the frequency of appearance of chromatin features in four-features simplified models reaching at least 60% of the full model accuracy, we found that features constituting the bootstrap-Lasso simplified model were clearly overrepresented in the feature appearance profile (Figure 2D).

Collectively, these results highlight key combinations of HMs and interactions thereof that harbor most of the information about replication timing. These combinations are indispensable to achieve faithful predictions and likely to reflect regulatory principles conserved across species. Particularly, monomethylation of H3K36 by the yeast Set2 methyltransferase has been shown to regulate the time of Cdc45 association with origins. Cdc45 is recruited to replication origins at the time of initiation and this binding event is delayed in Set2 mutants, suggesting a direct involvement of H3K36me1 in replication initiation [55]. Histone hyper-acetylation marks active origins of the *Drosophila* chorion loci [28] and H4K8ac colocalizes with ORC at these developmentally regulated genomic regions. Chorion origin activity can be altered by tethering of the histone deacetylase Rpd3 or of the acetyltransferase Chameau (the ortholog of human MYST2/HBO1), which reduces and increases origin firing, respectively [28]. In addition, recent work indicated histone hypoacetylation as a requirement for maintaining late replication timing of constitutive heterochromatin [56], supporting a view in which histone acetylation levels modulate origin activity. The Ubiquitination of H2B by the ubiquitin ligase Bre1 plays multifaceted, transcriptional dependent as well as independent roles at chromatin. The mark is mostly euchromatic and has been shown to be required for efficient transmethylation of H3 at positions K4 and K79 [57]. Very recent work implicated H2BUb1 in yeast DNA replication [58], where the mark promotes nucleosome assembly and their stability behind advancing replication forks. Although our results may seem to contradict these findings, the impact of a variable on replication timing can be uncoupled from its role during the DNA replication process *per se*. Interestingly, H2BUb1 was shown to modulate the overall chromatin structure by inducing nucleosome stability and mediating chromatin compaction, in contrast to its supposed role in opening up chromatin [59]. Nucleosome stabilization, in turn, can result in transcriptional repression and a global increase of H2Bub1 levels has been shown to impede cell growth in yeast [59]. Thus, we propose a negative effect of H2BUb on replication timing of euchromatin, where H2BUb enriched regions are characterized by reduced accessibility and more stable nucleosomes. In addition, the two interaction terms involving H2BUb, namely H2BUb:H3K36me1 and H2BUb:H3K79me1, suggest a hierarchy whereby nucleosome stability exerts a dominant effect over the presence of activating marks. Alternatively, these pairwise interactions might indicate a role of Bre1-Set2 and Bre1-Dot1 complexes in delaying euchromatic origin firing. Finally, as the H2BUb antibody used to generate the H2BUb ChIP-chip profile is not specific for mono-ubiquitination, polyubiquitylation of H2B might also be responsible for the inferred effect of H2BUb on replication timing. In yeast, extensive H2B polyubiquitylation occurs with at least two distinct modes, Bre1-dependent and independent, suggesting distinct, yet not elucidated, biological functions [57].

### Chromatin feature levels at promoters enable faithful prediction of the whole genome replication timing profile

**Figure 3:**
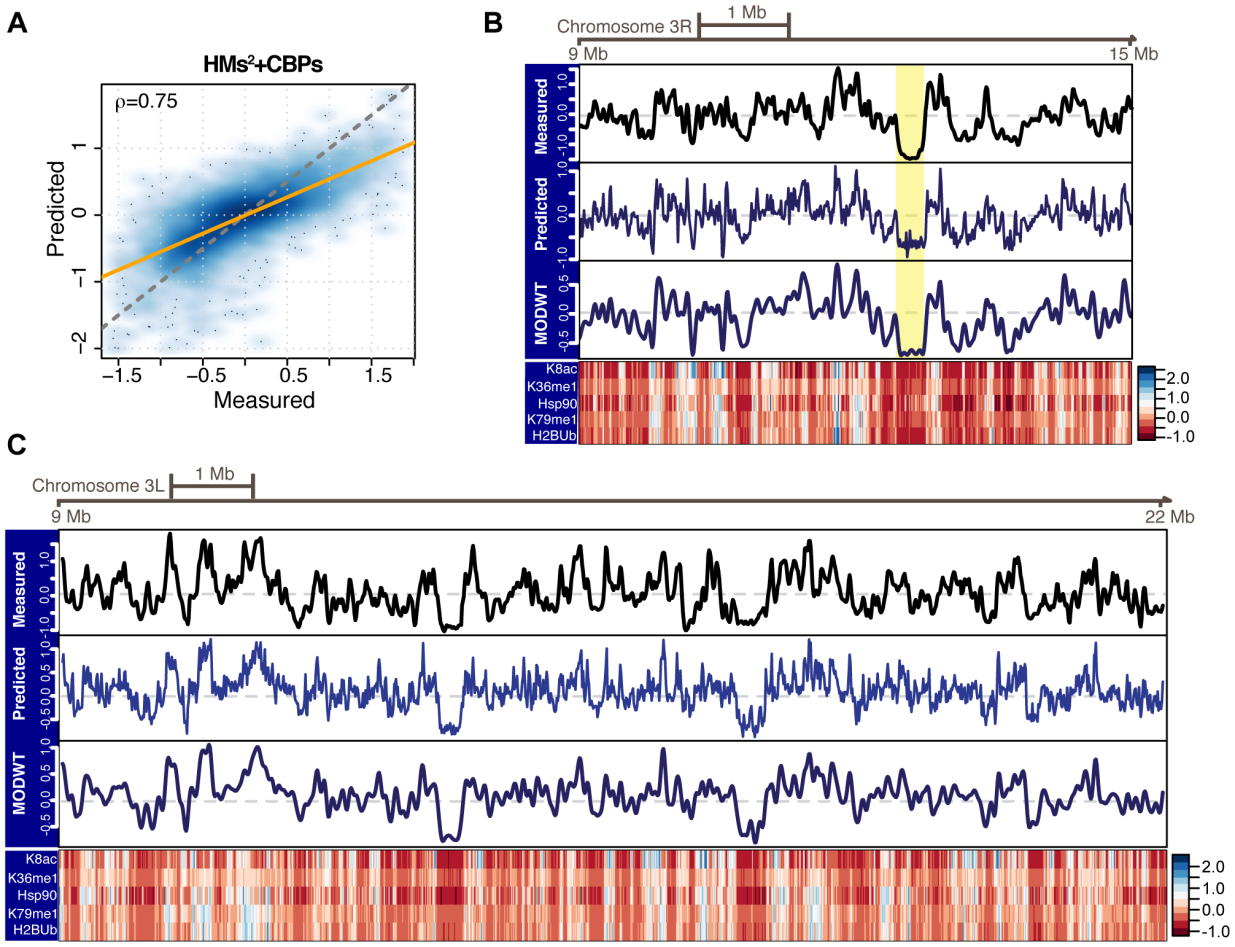
Predicting the replication timing profile of the *Drosophila* S2 cells genome. (A) Predicted versus experimentally measured replication timing of the *Drosophila* S2 cells genome represented as smoothed color density scatter plot. Model predictions were generated using the Lasso model based on CBPs and second-order interactions between HMs (HMs^2^+CBPs) and trained at promoters. Prediction accuracy is Pearson correlation coefficient. The orange line indicates the model fit, whereas the dashed gray line indicates the bisector 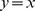. (B,C) Measured (top track) and predicted (middle and bottom track, see Methods) replication timing profiles along 6 Mb and 12 Mb of chromosomes 3R (B) and 3L (C), respectively. A color gradient representation of feature signals is shown at the bottom for chromatin features within the bootstrap-Lasso simplified model (K8ac = H4K8ac; K36me1 = H3K36me1 and K79me1 = H3K79me1). The yellow rectangle in B highlights the genomic position of the Bithorax Complex.

To assess whether the combinations of chromatin features learnt at promoters allow accurate prediction of the genome-wide replication timing profile, we segmented the *Drosophila* genome in 10 kb bins and computed feature levels therein (see Methods). Then, we used the Lasso model based on CBPs and pairwise interactions between HMs trained at promoters to evaluate its accuracy in predicting the whole genome replication timing profile of S2 cells. Interestingly, we found that promoter-proximal combinations of chromatin features enable accurate genome-wide predictions (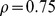, Figure 3A), with comparable prediction accuracies between individual chromosome arms (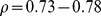, Supplementary Figure S7). These values are comparable to the accuracy obtained in promoter regions (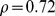, Figure 1E). Consistently with these results, the bootstrap-Lasso simplified model was able to predict the whole genome replication timing profile of S2 cells with an accuracy of 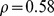 (Supplementary Figure S8), the same value exhibited at promoters. These results indicate that combinations of chromatin features with regulatory potential for replication timing can be generalized to the whole genome and are therefore not confined to promoter-proximal regions.

Given the good agreement between experimentally determined and inferred values, we visually compared measurements and predictions as a function of their genomic position, as shown in Figure 3B,C for 6 Mb and 12 Mb of chromosomes 3R and 3L, respectively. This visualization allows us to further evaluate model predictions. First, although Lasso does not account for the spatial organization of DNA replication timing and of HMs, yielding predictions that are more noisy than measured values, the overall structure of the measured replication timing profile was faithfully recapitulated by the inferred one. Second, denoising of predicted values using adaptive smoothing (see Methods) further increased the correlation between measured and predicted values genome-wide (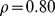) and made their similarity even more striking (Figure 3B,C). This result does not strongly depend on the degree of smoothing, as predicted profiles smoothed at 20 (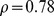), 40 (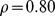) and 80 (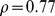) kb resolution similarly correlate with measured replication timing values. Third, early-to-late and late-to-early transition zones, which coincide with boundary elements separating distinct chromosomal domains such as those flanking the late replicating *Drosophila* Bithorax complex [60] (Figure 3B, yellow rectangle), were accurately inferred by the model. However, we systematically investigated whether the prediction accuracy was uniform across different classes of genomic regions or whether some regions could be predicted with higher accuracy than others. To this purpose, we identified timing transition regions (TTRs) and replication domains using a circular binary segmentation algorithm (see [4] and Methods for details), and determined gene dense and poor regions using a two-state Hidden Markov Model (see Methods). We found that prediction accuracies were higher in replication domains (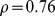) and gene poor regions (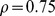) than in TTRs (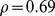) and gene dense regions (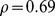), respectively (Supplementary Figure S9). The reduced prediction accuracy at TTRs might depend on the fact that these regions are devoid of replication origins and other chromatin features, and result from passive unidirectional replication fork movement [17]. In contrast, as the *Drosophila* genome is rather compact, it is plausible that feature averaging in gene dense bins partially reduces prediction accuracy in these regions. Alternatively, as our model globally underestimated early replication timing peaks and as gene density positively correlates with replication timing [36], a subset of chromatin determinants of early origin firing might not yet be part of the profiled CBPs and HMs and remains to be elucidated. Since CBPs are generally characterized by narrower peaks as compared to HMs and hence contribute more locally to replication timing predictions, it is likely that the missing features will correspond to CBPs exhibiting preferential binding to open chromatin.

### Combinations of histone modifications predict DNA replication timing across different cell types

**Figure 4:**
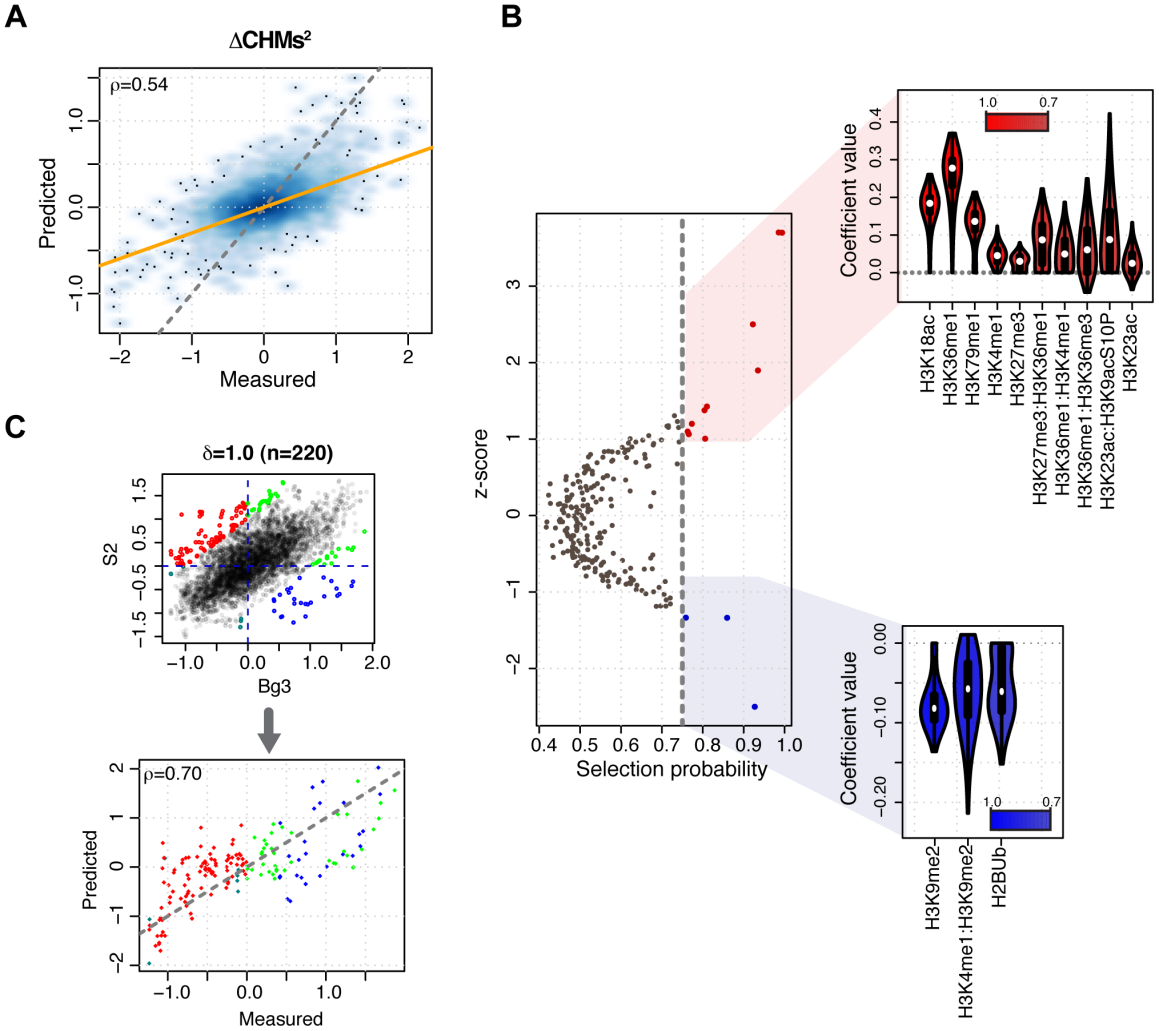
Histone modification levels predict replication timing across different cell types. (A) Predicted versus experimentally measured differences in replication timing between S2 and Bg3 cells unique promoters (S2-Bg3) represented as smoothed color density scatter plot. Model predictions were generated using differences in HMs levels and their pairwise interactions for a subset of HMs that were profiled in both S2 and Bg3 cell lines (CHMs). The orange line indicates the model fit, whereas the dashed gray line indicates the bisector 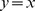. (B) Scatter plot of model features according to their *z*-scores and bootstrap-Lasso selection probabilities (*p*). Features with 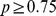 are colored in red (positive coefficient values) or blue (negative coefficient values) and their coefficient distributions are shown on the right as violin plots. Features are ranked by decreasing selection probabilities. (C, top) Replication timing of S2 cells promoters versus Bg3. Differentially replicating promoters are color-coded according to the quadrant (delimited by dashed blue lines) they belong to (red: early replicating in S2 and late replicating in Bg3; green: early in both S2 and Bg3; blue: late in S2 and early in Bg3, aqua: late in both S2 and Bg3). A total of 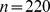 promoters exhibit a log fold change greater than or equal to 1 (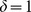). (C, bottom) Experimentally determined replication timing in Bg3 versus predictions generated by a model based on pairwise interactions between CHMs in S2 cells. Prediction accuracy is Pearson correlation coefficient. The dashed gray line indicates the bisector 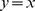.

We have shown that combinatorial modeling of chromatin features can accurately predict DNA replication timing in S2 cells. However, the chromatin landscape varies between cell types, and similarly, replication timing is a cell-type specific epigenetic feature [5]. Previous work from Eaton *et al.* [15] showed that clusters of chromatin features are predictive for changes in early origin strength across cell types. Thus, we focused on promoters and asked whether differences in the chromatin landscape between two cell types can explain the corresponding differences in their replication timing. Besides for S2 cells, genome-wide DNA replication timing and chromatin feature profiles are available for *Drosophila* Bg3 and Kc cell lines from modENCODE. The replication timing profiles of these two cell lines are highly correlated to each other and with the replication timing of S2 cells (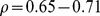 at promoters, Supplementary Figure S10). As the number of HMs profiled in both S2 and Bg3 is larger than those in common between S2 and Kc, we considered 21 HMs that were profiled in the former two cell lines (termed CHMs, in Common Histone Modifications, and listed in Methods) for further analyses. First, we assessed the predictive power of CHMs on replication timing at promoters in S2 and Bg3 cells, respectively. For each cell line, we trained a Lasso model based on the corresponding levels of CHMs and their pairwise interactions and obtained fairly accurate predictions in both cell types (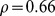 and 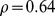 in S2 and Bg3 cells, respectively; Supplementary Figure S11). These models are cell line specific as their accuracy in predicting unmatched replication timing profiles is significantly lower than the one achieved on the matched profile (data not shown). We next investigated whether differences in replication timing between S2 and Bg3 cells can be predicted from differences in CHMs (ΔCHMs) levels between these two cell lines. Therefore, we used ΔCHMs (S2-Bg3) and their pairwise interactions to predict differential replication timing (S2-Bg3, see Methods) and found that the model was able to achieve a prediction accuracy of 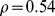 (Figure 4A). Although this result indicates that inferring differences in replication timing is more challenging than inferring the timing *per se*, differences in HMs levels bear a fair predictive power on differential replication timing. Next, we investigated feature importance in predicting differential replication and estimated feature selection probabilities using bootstrap-Lasso as described before. We found that H3K18ac, H3K36me1 and its interactions with H3K27me3, H3K4me1 and H3K36me3, as well as H3K79me1 are selected with high probability and predictive for earlier replication in S2 than Bg3 cells (positive differences, Figure 4B). On the other hand, H3K9me2 and its interaction with H3K4me1, along with H2BUb levels, are stable predictors for later replication timing values in S2 than Bg3 cells (negative differences, Figure 4B). Overall, this analysis revealed that cell-type-specific differences in HMs are more predictive for differences in replication timing than cell-type-specific differences in interactions between HMs.

Finally, we narrowed our attention to differentially replicating promoters (DRPs) between S2 and Bg3 cell lines and asked whether the Lasso model trained on CHMs levels in S2 cells (Supplementary Figure S11A) can predict the replication timing of DRPs in Bg3 cells. The set of DRPs was defined using three different fold change cutoffs at the high end of the overall fold changes in replication timing (Figure 4C, see Methods). Notably, we found that the model based on S2 data was able to predict the replication timing of DRPs in Bg3 cells with high accuracy for all three cutoffs (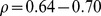, Figure 4C and Supplementary Figure S12). Prediction accuracies did not vary significantly upon further increase of the cutoff. Taken together, these results indicate that combinations of HMs allow a general, cell-type independent description of the relationship between replication timing and chromatin.

### Conclusions and perspectives

We systematically investigated the relevance of combinatorial HM patterns for DNA replication timing in *Drosophila* using Lasso. Developed on linear combinations of chromatin features from a comprehensive collection of HMs and CBPs profiles, our model quantitatively predicts replication timing with high accuracy genome-wide and across cell types. Our results show that combinations of HMs and their pairwise interactions are key in achieving accurate predictions, suggesting that combinatorial HM patters might indeed contribute to the regulation of DNA replication timing. However, it is important to notice that our data and analysis do not allow us to infer causality. Therefore, our description of the relationship between chromatin features and replication timing is a correlative one. In addition, there is a remaining 48% of variation in DNA replication timing that is not explained by our model. Accurate estimates of the maximal fraction of the observed variation in replication timing that could theoretically be explained by the model - e.g. following the recent approach proposed by [61] - were not possible in our framework due to lack of biological replicates for a subset of features and would have nevertheless been challenged by data integration across different laboratories and platforms. Unexplained variation can be possibly due to missing key features, presence of nonlinearities in the modeled relationship and existence of additional factors other than CBPs and HMs, such as the chromatin architecture, contributing to replication timing regulation. Although it is plausible that key determinants of DNA replication timing have not yet been profiled, it is unlikely that this aspect alone could entirely fill the gap. Since the regulatory mode of replication timing has not yet been fully elucidated, we hypothesized that a nonlinear relationship between chromatin landscape and replication timing could explain, at least partially, the remaining variation in DNA replication timing. We tested this hypothesis by using multivariate adaptive regression splines (MARS) [62], a flexible non-parametric regression technique based on piecewise linear basis functions which can also be adopted to estimate feature importance. However, MARS prediction accuracies were comparable to Lasso irrespective of model complexity (Supplementary Table S1), indicating that the relationship between chromatin feature levels and replication timing is well modeled by a linear function. For consistency, performances of the Lasso and MARS fits were also tested and confirmed using a second, independently generated, genome-wide replication timing profile in S2 cells [37] (Supplementary Table S1).

Through feature importance analyses, we identified a minimal set of six terms whose prediction accuracy reaches 80% of the full model accuracy. Remarkably, all elements within this set were selected by the MARS fit, with H4K8ac, H3K36me1, H2BUb:H3K36me1, H2BUb and Hsp90 indicated as the five most important terms. Besides demonstrating the necessity of these features to achieve high prediction accuracy, our results contribute experimentally testable, putative elements of a combinatorial HM pattern for DNA replication. In addition, availability of genome-wide profiles for these features in the same human or mouse cell line will enable to assess whether their predictive power is conserved across species. Finally, experimental investigation of our simplified model terms might unravel the mechanistic basis of their connection to DNA replication, and thereby, shed light on the regulatory mode of this fundamental cellular process.

## Materials and Methods

### Data

Genome-wide replication timing profiles of *Drosophila* S2 and Bg3 cell lines (GEO accession numbers GSE17280 and GSE17281, respectively) were generated by Eaton et al. [15] using Agilent tiling arrays. Normalized smoothed *M*-values were used for the analysis. ChIP-Seq profiles of CBPs and HMs in S2 cells were downloaded as raw data in sra format from the Short Read Archive (SRA) or fetched from the Gene Expression Omnibus (GEO). Matched input datasets were downloaded where available. ChIP-chip profiles were downloaded from the modENCODE [38] data warehouse. Normalized smoothed *M*-values as provided by modENCODE were used for the analysis. If a feature was profiled more than once, only one profile was considered by taking into account antibodies characterization and technological platforms and by prioritizing deep sequencing based profiles. Pairwise Pearson's correlations *ρ* of feature signals at promoters were computed between the selected profile and all possible alternatives and typically 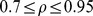. A list of the datasets included in the analysis is provided in Supplementary Tables S2 and S3 for CBPs and HMs, respectively.

### Selecting genomic regions for analysis

Chromosome arms 2L, 2R, 3L and 3R were considered for the analysis. Chromosomes 4 and X were excluded due to special chromatin characteristics. Specifically, the single male X chromosome is hyperacetylated on H4K16 [63] and completes replication significantly earlier than the autosomes in male cell lines [37] whereas the fourth chromosome is predominantly heterochromatic and exhibits a high-transposon density [65]. For the prediction of DNA replication timing of promoters, Ensembl gene annotations were downloaded from biomart (www.biomart.org, genome assembly BDGP 5.12) using the R package biomaRt [64]. Promoter regions were defined as 1 kb windows centered on unique transcription start sites (TSS) in order to limit ambiguous assignment of chromatin feature signals to promoters. We defined a TSS as unique if no other TSS was annotated within the 1 kb genomic region flanking the TSS, regardless of the strand. A total of 7552 unique promoters was then considered for the analysis. For the prediction of genome-wide replication timing, the *Drosophila* genome was segmented into bins of width 10 kb. A total of 9663 bins was used for the analysis.

### Scoring of chromatin features

ChIP-Seq data in sra format were first converted to fastq format using the NCBI Short Read Archive Toolkit and subsequently aligned to the Flybase *Drosophila melanogaster* dm3 reference genome assembly r5.22 using Bowtie 0.12.8 with parameters [-n 2 -k 1, –best and -M 100]. Matched input datasets were aligned using the same parameters. The alignment output was converted from SAM to BAM format using SAMtools 0.1.18 and BAM files were imported in R using Rsamtools (Morgan, M. and Pagès, H., Rsamtools: Binary alignment (BAM), variant call (BCF), or tabix file import, R package version 1.8.6). Feature signals in both promoters and genomic bins were estimated as follows. Given a sample dataset *S* and an input dataset *I* the feature enrichment *M* of *S* relative to *I* within a given region of interest *R* was computed using available *D* replicates as follows. Let 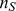 and 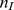 be the library size of *S* and *I*, respectively and *p* an integer pseudocount used to avoid undefined values in logarithmic transformations (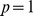 in this analysis). Then, define 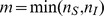. Finally, let 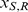 and 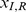 be the number of short reads entirely aligning within *R* for sample and input datasets, respectively. For each replicate *d* we then computed:
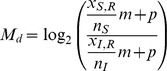
and defined the feature enrichment as 
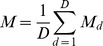
. For ChIP-chip datasets, the feature signal was computed as mean smoothed *M*-value within *R*. Similarly, the replication timing of *R* was computed as the average replication timing value of probesets mapping entirely within *R*.

### Hierarchical clustering of chromatin features at promoters

Hierarchical clustering of chromatin features at promoters was performed using a correlation-based dissimilarity measure between feature signals at promoters. Given two profiles 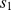 and 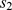, their dissimilarity *d* was computed as 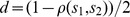, where *ρ* denotes the Pearson's correlation coefficient.

### Predicting DNA replication timing using Lasso

The relationship between DNA replication timing and chromatin features was modeled using Lasso. Briefly, let 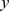 be the dependent variable (DNA replication timing), 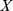 be the 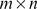 enrichment matrix where 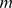 is the number of promoters and 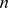 the number of independent variables (chromatin features and when considered, their interaction terms) and 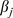 the 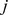-th linear model coefficient associated to the 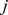-th independent variable. The Lasso parameters 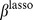 are then estimated as:
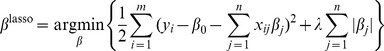
where the first term corresponds to the residual sum of squares commonly minimized by multiple linear regression models and where the second term 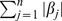 is the 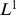 Lasso penalty that is tuned by the regularization parameter 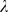.

To fit the model, the set of 7552 unique promoters was randomly partitioned into two sets 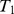 (5000 promoters) and 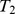 (2552 promoters). The model was trained on 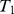 with ten-fold cross validation. The cross-validated mean squared error (CV-MSE) as a function of 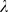 was used to inspect the model fit. The value of 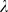 minimizing the CV-MSE was used to predict the replication timing of the test set 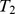. The Pearson correlation coefficient between measured and predicted continuous replication timing values on 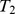 was used to determine the model accuracy.

### Deriving simplified models

Simplified models were obtained using three different approaches: i) By analyzing the coefficients of the Lasso model based on CBPs and interactions between HMs along the 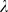-path used to fit the model. Only models leading to a prediction accuracy of at least 75% of the prediction accuracy achieved by the full model were considered; ii) By performing stability analysis of model coefficients (see below); iii) By generating all possible combinations of two (4 371), three (134 044) and four (3 049 501) features and training a multiple linear regression model based on each combination following the same procedure described above for the Lasso model fit. The Bayesian Information Criterion was used to account for model complexity and assess whether increasing the number of features was still beneficial for the model fit.

### Computing feature selection probabilities

Stability analysis of model coefficients was performed essentially as described in [48]. Feature selection probabilities (normalized frequencies of non-zero coefficients) were computed using bootstrap-Lasso. Briefly, a Lasso model based on CBP levels and interactions between HMs was trained with ten-fold cross validation using all 7552 unique promoters. The values of the regularization parameter 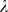 yielding an empty model (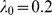) and an almost full model (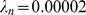) were used to construct a sequence 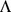 of 100 values ranging from 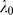 to 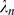 with constant ratios between consecutive elements. This sequence was then used to fit 100 Lasso models on 100 bootstrap samples of 7552 promoters. Model coefficients were stored for each value of 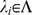 and for each fitted model. Finally, for each chromatin feature the number of non-zero coefficients was summed and normalized to the total number of recorded coefficients. Normalized values represent the estimated selection probabilities.

### Estimating the significance of selected model features

The overall significance of H3K36me1, H4K8ac, H2BUb and Hsp90 in predicting replication timing was estimated using a bootstrap-based approach. For each feature, 100 bootstrap samples of 7552 promoters were generated and partitioned into 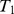 and 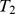 as above. Each 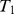 sample was used to train a Lasso model based on CBPs and pairwise interactions of HMs but lacking all model terms involving the selected feature with ten-fold cross validation. Model accuracies (PCC) on 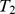 were recorded and compared to the accuracies achieved by full models trained on the same bootstrap samples using a two-sided Wilcoxon rank sum test.

### Smoothing of predicted DNA replication timing profiles

Adaptive smoothing of predicted genome-wide replication timing profile was performed using a maximum overlap discrete wavelet transform (MODWT). In pratice, we used the R package waveslim (Whitcher,B., waveslim: Basic wavelet routines for one-, two- and three-dimensional signal processing, R package version 1.7.1) with la8 wavelet filter, J = 2 and reflecting boundaries.

### Identifying timing transition regions

Timing transition regions (TTRs) were identified using the circular binary segmentation algorithm implemented in the R package DNAcopy (Venkatraman,E.S. and Olshen,A., DNAcopy: DNA copy number data analysis, R package version 1.32.0) according to [4]. Replication timing of the 9663 *Drosophila* genomic bins at 10 kb resolution was provided as input and a 30 kb window centered on each identified domain boundary was used to define a TTR. Visual inspection of the segmented replication timing profile was performed and verified accurate recognition of TTRs.

### Determining gene dense and gene poor regions

Gene density along the *Drosophila* genome was computed using Ensembl gene annotations at a 10 kb resolution and used to classify gene poor and gene dense regions by learning a two-state Hidden Markov Model (HMM). The HMM was fit using the Baum-Welch algorithm implemented in the R package RHmm (Taramasco,O. and Bauer,S., RHmm: Hidden Markov Models simulations and estimations, R package version 2.0.3) and the optimal hidden states sequence was computed using the Viterbi algorithm.

### Predicting DNA replication timing across cell types

A subset of 21 HMs (termed in Common Histone Modifications, CHMs) that have been profiled by modENCODE in both S2 and Bg3 cell lines was considered. This set includes the following features: H1, H2BUb, H3K18ac, H3K23ac, H3K27ac, H3K27me2, H3K27me3, H3K36me1, H3K36me3, H3K4me1, H3K4me3, H3K79me1, H3K79me2, H3K9acS10P, H3K9ac, H3K9me1, H3K9me2, H3K9me3, H4K16ac, H4K20me and H4. Feature scoring and computation of DNA replication timing at unique promoters were performed as described above. The predictive power of CHMs on replication timing of S2 and Bg3 promoters was evaluated using Lasso models based on second-order interactions between CHMs. For each cell line, a model was trained on 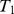 with ten-fold cross validation using the corresponding CHMs levels. Model accuracy (PCC) was determined on 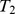.

To test whether differential CHMs between cell lines can predict differential replication timing (S2-Bg3, Δ*t*), we computed differences in CHMs levels between S2 and Bg3 cells (ΔCHMs) and used them to predict Δ*t* through a Lasso model with pairwise interactions. To predict the replication timing of differentially replicating promoters (DRPs) in Bg3 cells, we defined DRPs based on log fold change differences between S2 and Bg3 promoters using three increasing cutoff values (0.8, 0.9 and 1.0). The Lasso model introduced above to evaluate the predictive power of CHMs in S2 cells was then applied to infer the replication timing of DRPs in Bg3 cells.3

### Availability of R scripts

All analyses were performed using R 3.0.0 (R Core Team, R: A Language and Environment for Statistical Computing, http://www.R-project.org). Custom R scripts are available from https://github.com/FedericoComoglio/ToR.

## Supporting Information

Figure S1 **Hierarchical clustering of chromatin feature levels at promoters.** (A) Chromatin binding proteins (B) Histone modifications. Correlation-based dissimilarities (see Methods in the main text) are colored according to the top right color key.
(TIF)

Figure S2 **Individual predictive power of chromatin features.** Predictive power of individual chromatin feature levels on replication timing at promoters in S2 cells. Histone modifications (top) are separated from chromatin binding proteins (bottom) by a red dashed line. Gray bars represent the average model accuracy as PCC (Pearson's correlation coefficient) obtained from 10-fold cross-validation of a univariate linear model. Error bars represent standard deviations.
(TIF)

Figure S3**Combinatorial predictive power of chromatin features (I).** (A) Cross-validated mean squared error (CV-MSE) as a function of the regularization parameter (log_10_(*λ*)) for different Lasso models trained with ten fold cross-validation. The average CV-MSE is reported as solid line, with minimum and maximum CV-MSE drawn as dashed lines. A vertical line reaching a CV-MSE curve indicates the value of *λ* that was used to generate predictions from the corresponding model. The different sets of features used for model training are indicated in the legend. (B–E) Predicted versus experimentally measured replication timing of the test set represented as smoothed color density scatter plot. Model predictions were generated using the indicated sets of features. Prediction accuracies are Pearson correlation coefficients. Orange lines indicate the model fit, whereas dashed gray lines indicate the bisector 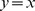.(TIF)

Figure S4**Combinatorial predictive power of chromatin features (II).** (A–B) Predicted versus experimentally measured replication timing of the test set represented as smoothed color density scatter plot. Model predictions were generated using HMs and CBPs (HMs+CBPs, A) and second-order interaction terms of HMs and CBPs, encompassing pairwise interactions between HMs, CBPs and interactions between HMs and CBPs ((HMs+CBPs)^2^, B). Prediction accuracies are Pearson correlation coefficients. Orange lines indicate the model fit, whereas dashed gray lines indicate the bisector 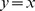.(TIF)

Figure S5 **Sequencing depth analysis of ChIP-Seq-based chromatin features.** (A) Sequencing depth, expressed as total number of aligned reads, for each ChIP-Seq profile included in this work. Features are ranked by decreasing coverage values and Hsp90 is highlighted in blue. (B) Individual predictive power of ChIP-Seq-based chromatin features as a function of their sequencing depth. Prediction accuracies are Pearson correlation coefficients. The orange lines indicates the fitted univariate linear regression model fit.
(TIF)

Figure S6 **Prediction accuracies of simplified models obtained through exhaustive model search.** (A) The value of the Bayesian Information Criterion (BIC) for the best simplified model (i.e. the model yielding the highest prediction accuracy (Pearson's correlation coefficient, PCC) in each group of one-, two-, three- and four-feature simplified models. (B) Boxplot of prediction accuracies for all simplified models within the same groups of models.
(TIF)

Figure S7**Predicting the replication timing of individual chromosome arms of the *Drosophila* S2 cells genome.** Predicted versus experimentally measured replication timing of the *Drosophila* S2 cells genome for individual chromosome arms: (A) 2L (B) 2R (C) 3L (D) 3R. Model predictions were generated using chromatin binding proteins and second-order interactions between histone modifications (HMs^2^+CBPs) from a model trained at promoters. Prediction accuracies are Pearson correlation coefficients. Orange lines indicate the model fit, whereas dashed gray lines indicate the bisector 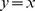.(TIF)

Figure S8**Prediction accuracy of the bootstrap-Lasso simplified model on the whole *Drosophila* S2 cells genome.** Predicted versus experimentally measured replication timing of the *Drosophila* S2 cells genome represented as smoothed color density scatter plot. Model predictions were generated using the six-features (H4K8ac, H3K36me1, H2BUb, H2BUb:H3K36me1, H2BUb:H3K79me1 and Hsp90) bootstrap-Lasso simplified model trained at promoters. Prediction accuracy is Pearson correlation coefficient. The orange line indicates the model fit, whereas the dashed gray line indicates the bisector 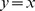.(TIF)

Figure S9**Evaluating prediction accuracies at different classes of genomic regions.** Predicted versus experimentally measured replication timing of the *Drosophila* S2 cells genome at: (A) timing transition regions (B) early/late replication domains (C) gene poor regions (D) gene dense regions. Model predictions were generated using chromatin binding proteins and second-order interactions between histone modifications (HMs^2^+CBPs) from a model trained at promoters. Prediction accuracies are Pearson correlation coefficients. Orange lines indicate the model fit, whereas dashed gray lines indicate the bisector 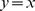.(TIF)

Figure S10 **Correlation of DNA replication timing profiles at promoters in S2, Kc and Bg3 cell lines.** Pairwise smoothed color density scatter plots between DNA replication timing of promoters in S2, Kc and Bg3 cell lines. Upper triangular entries are Pearson's correlation coefficients.
(TIF)

Figure S11**Evaluating the predictive power of HMs levels in common between S2 and Bg3 cells.** Predicted versus experimentally measured replication timing of the test set represented as smoothed color density scatter plot. Model predictions were generated based on second-order interactions between HMs levels in S2 cells (A) and Bg3 cells (B), using only a subset of HMs that were profiled in both cell lines (CHMs^2^). Prediction accuracies are Pearson correlation coefficients. Orange lines indicate the model fit, whereas dashed gray lines indicate the bisector 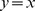.(TIF)

Figure S12**Predicting the replication timing of promoters that differentially replicate between S2 and Bg3 cells.** (A, left) Replication timing of S2 cells promoters versus Bg3. Differentially replicating promoters are color-coded according to the quadrant (delimited by dashed blue lines) they belong to (red: early replicating in S2 and late replicating in Bg3; green: early in both S2 and Bg3; blue: late in S2 and early in Bg3, aqua: late in both S2 and Bg3). A total of *n* = 528 promoters exhibit a log fold change greater than or equal to 0.8 (*δ* = 0.8). (A, right) Experimentally determined replication timing in Bg3 versus predictions generated by a model based on pairwise interactions between CHMs in S2 cells. Prediction accuracy is Pearson correlation coefficient. The dashed gray line indicates the bisector 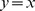. (B) Same as A, for *δ* = 0.9.(TIF)

Table S1 **Pearson's correlation coefficients between measured and predicted replication timing for different sets of chromatin features and both Lasso and MARS statistical models.** In addition, Lasso predictions on replication timing of S2 promoters are indicated for the model trained and tested using a second replication timing profile generated by Schwaiger *et al.* [37]. CBPs: chromatin binding proteins; HMs: histone modifications; HMs^2^+CBPs: CBPs and second-order multiplicative interactions between HMs; (HMs+CBPs)^2^: second-order multiplicative interaction terms of HMs and CBPs, encompassing pairwise interactions between HMs, CBPs and interactions between HMs and CBPs.
(PDF)

Table S2 **Summary of the chromatin binding protein profiles included in the analysis.** This table provides details and accession numbers of the chromatin binding protein profiles used for modeling.
(PDF)

Table S3 **Summary of the histone modification profiles included in the analysis.** This table provides details and accession numbers of the histone modification profiles used for modeling.
(PDF)
