# Supplementary figures and images for: Combinatorial Modeling of Chromatin Features Quantitatively Predicts DNA Replication Timing in Drosophila
Source: PLoS Comput Biol. 2014 Jan 23;10(1):e1003419. doi: 10.1371/journal.pcbi.1003419 (PMC3900380; doi:10.1371/journal.pcbi.1003419)

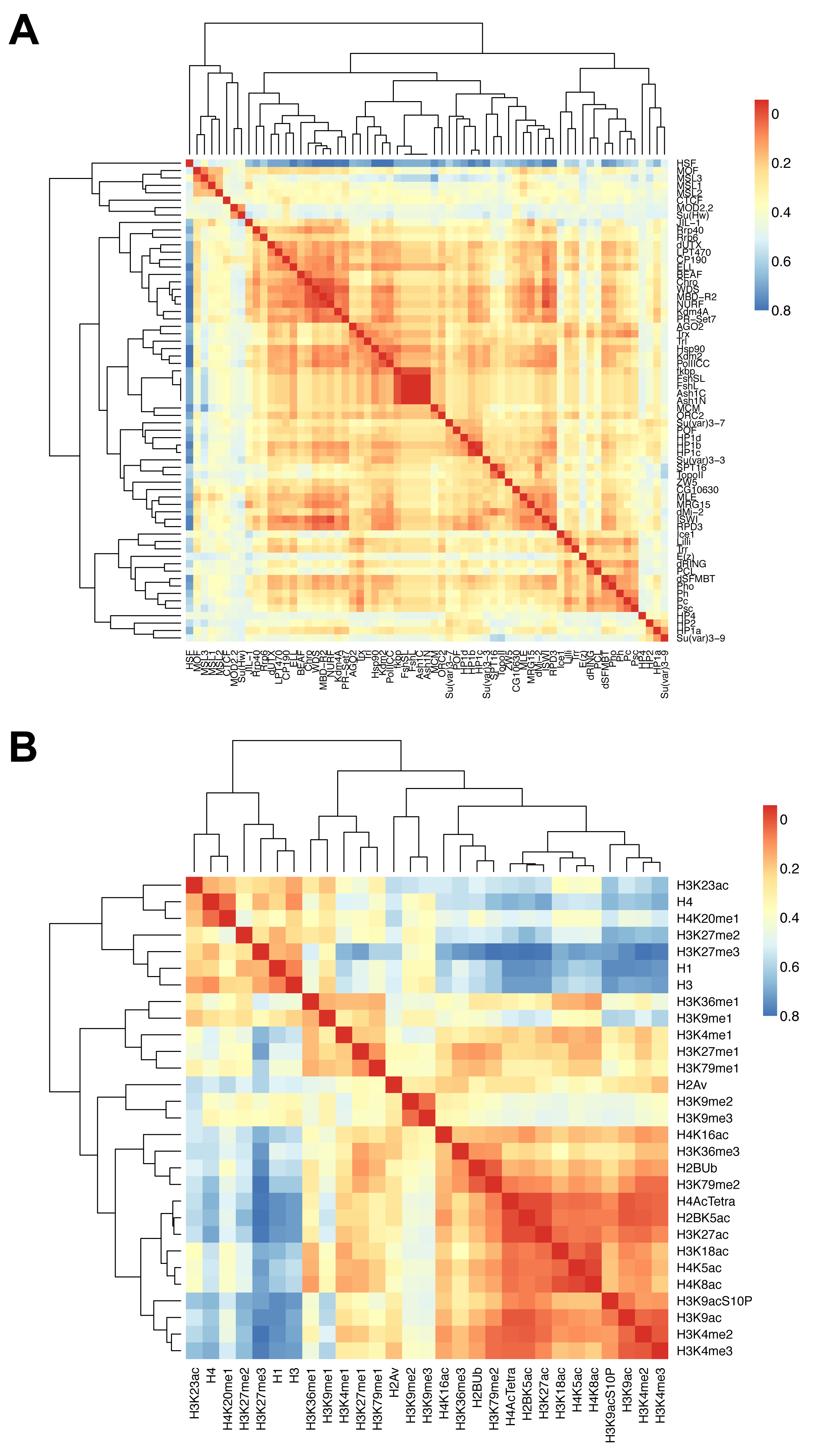

Supplement: Figure S1 — Hierarchical clustering of chromatin feature levels at promoters. (A) Chromatin binding proteins (B) Histone modifications. Correlation-based dissimilarities (see Methods in the main text) are colored according to the top right color key. (TIF) [file pcbi.1003419.s001.tif]

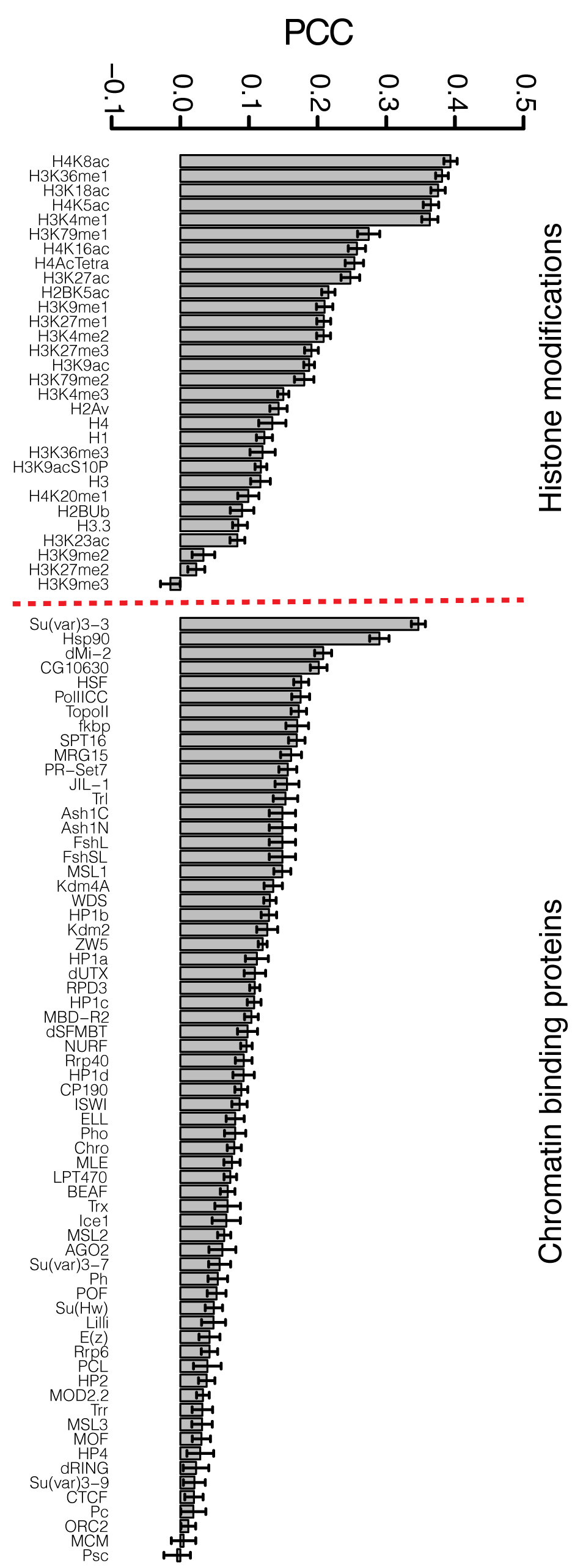

Supplement: Figure S2 — Individual predictive power of chromatin features. Predictive power of individual chromatin feature levels on replication timing at promoters in S2 cells. Histone modifications (top) are separated from chromatin binding proteins (bottom) by a red dashed line. Gray bars represent the average model accuracy as PCC (Pearson's correlation coefficient) obtained from 10-fold cross-validation of a univariate linear model. Error bars represent standard deviations. (TIF) [file pcbi.1003419.s002.tif]

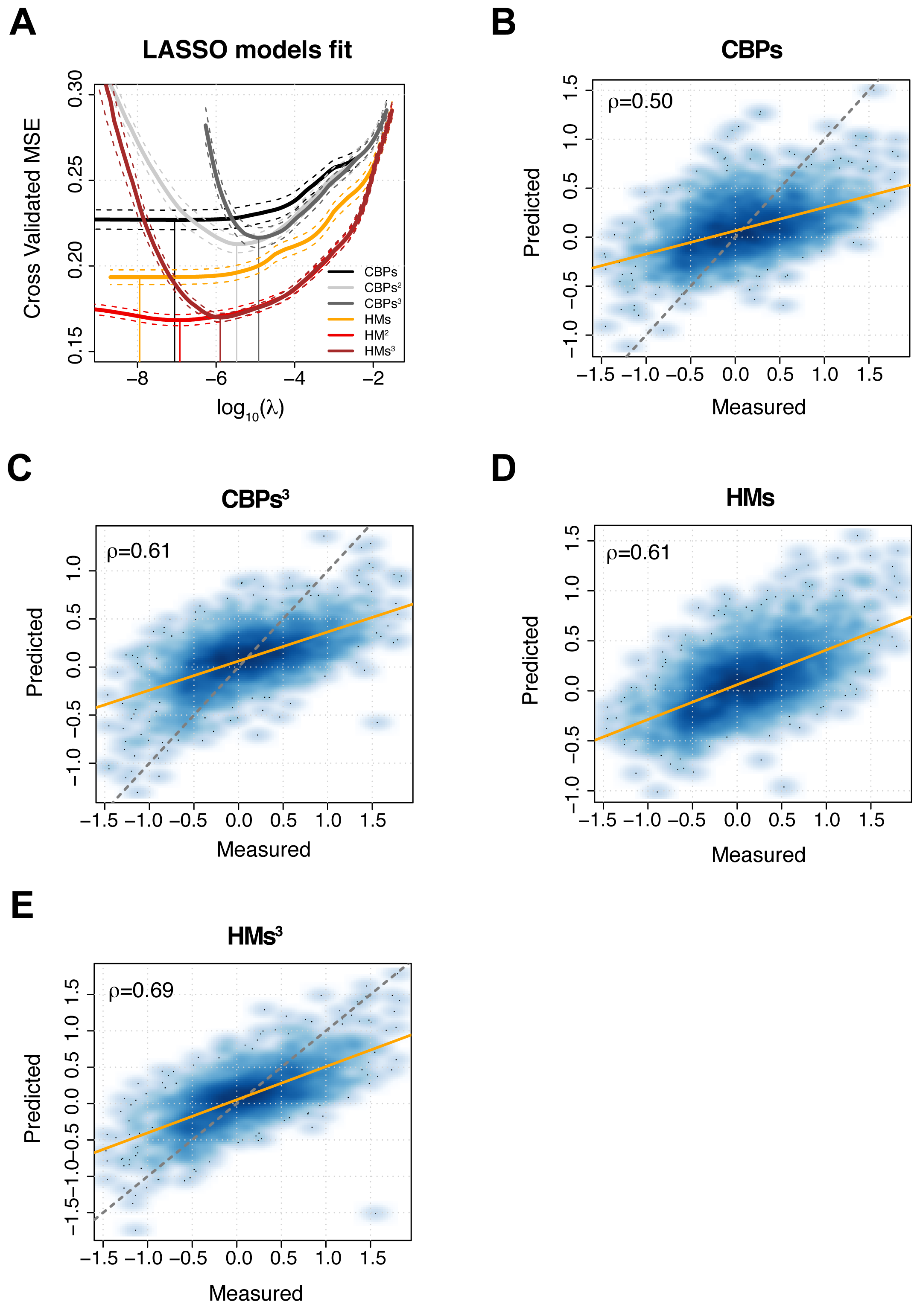

Supplement: Figure S3 — Combinatorial predictive power of chromatin features (I). (A) Cross-validated mean squared error (CV-MSE) as a function of the regularization parameter (log10(λ)) for different Lasso models trained with ten fold cross-validation. The average CV-MSE is reported as solid line, with minimum and maximum CV-MSE drawn as dashed lines. A vertical line reaching a CV-MSE curve indicates the value of λ that was used to generate predictions from the corresponding model. The different sets of features used for model training are indicated in the legend. (B–E) Predicted versus experimentally measured replication timing of the test set represented as smoothed color density scatter plot. Model predictions were generated using the indicated sets of features. Prediction accuracies are Pearson correlation coefficients. Orange lines indicate the model fit, whereas dashed gray lines indicate the bisector . (TIF) [file pcbi.1003419.s003.tif]

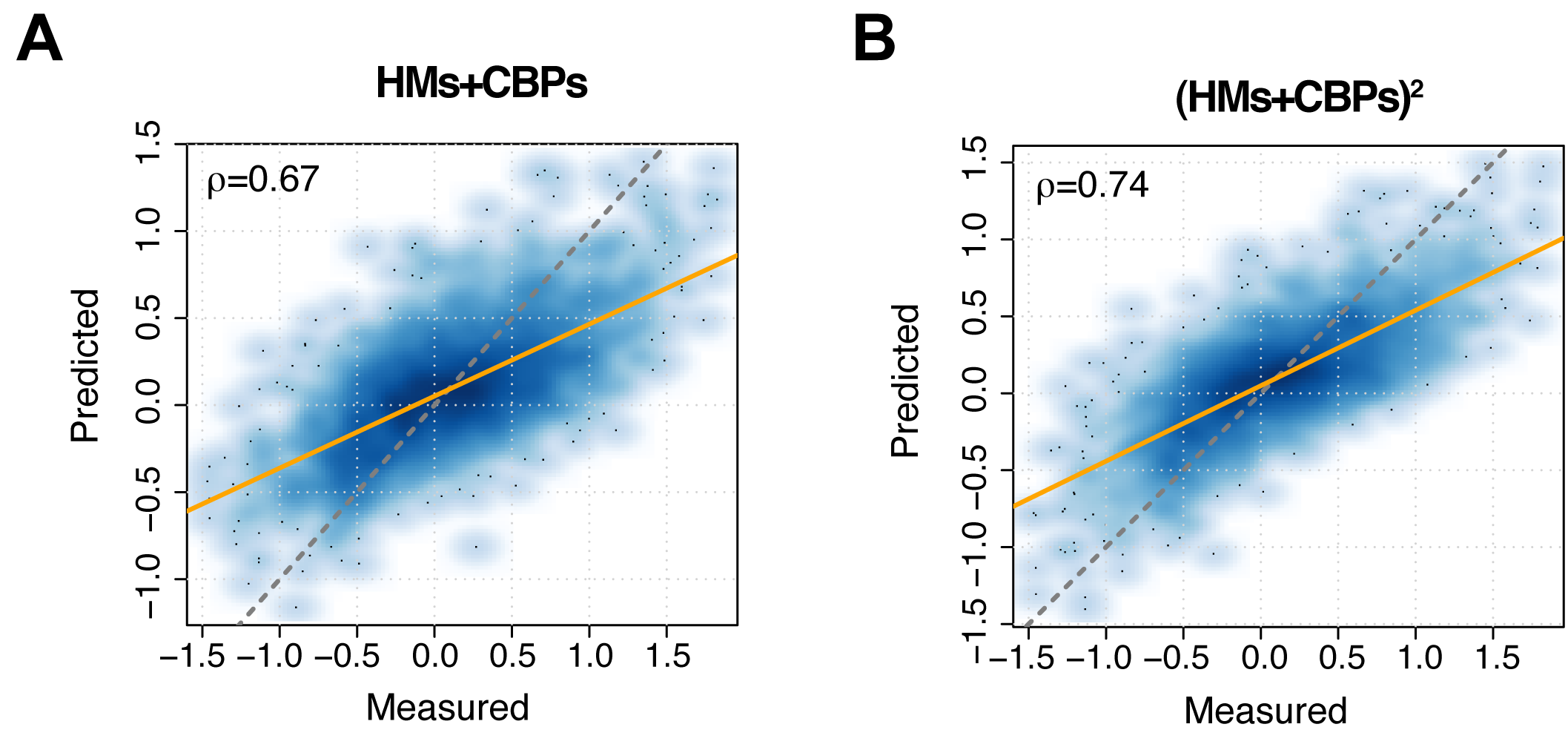

Supplement: Figure S4 — Combinatorial predictive power of chromatin features (II). (A–B) Predicted versus experimentally measured replication timing of the test set represented as smoothed color density scatter plot. Model predictions were generated using HMs and CBPs (HMs+CBPs, A) and second-order interaction terms of HMs and CBPs, encompassing pairwise interactions between HMs, CBPs and interactions between HMs and CBPs ((HMs+CBPs)2, B). Prediction accuracies are Pearson correlation coefficients. Orange lines indicate the model fit, whereas dashed gray lines indicate the bisector . (TIF) [file pcbi.1003419.s004.tif]

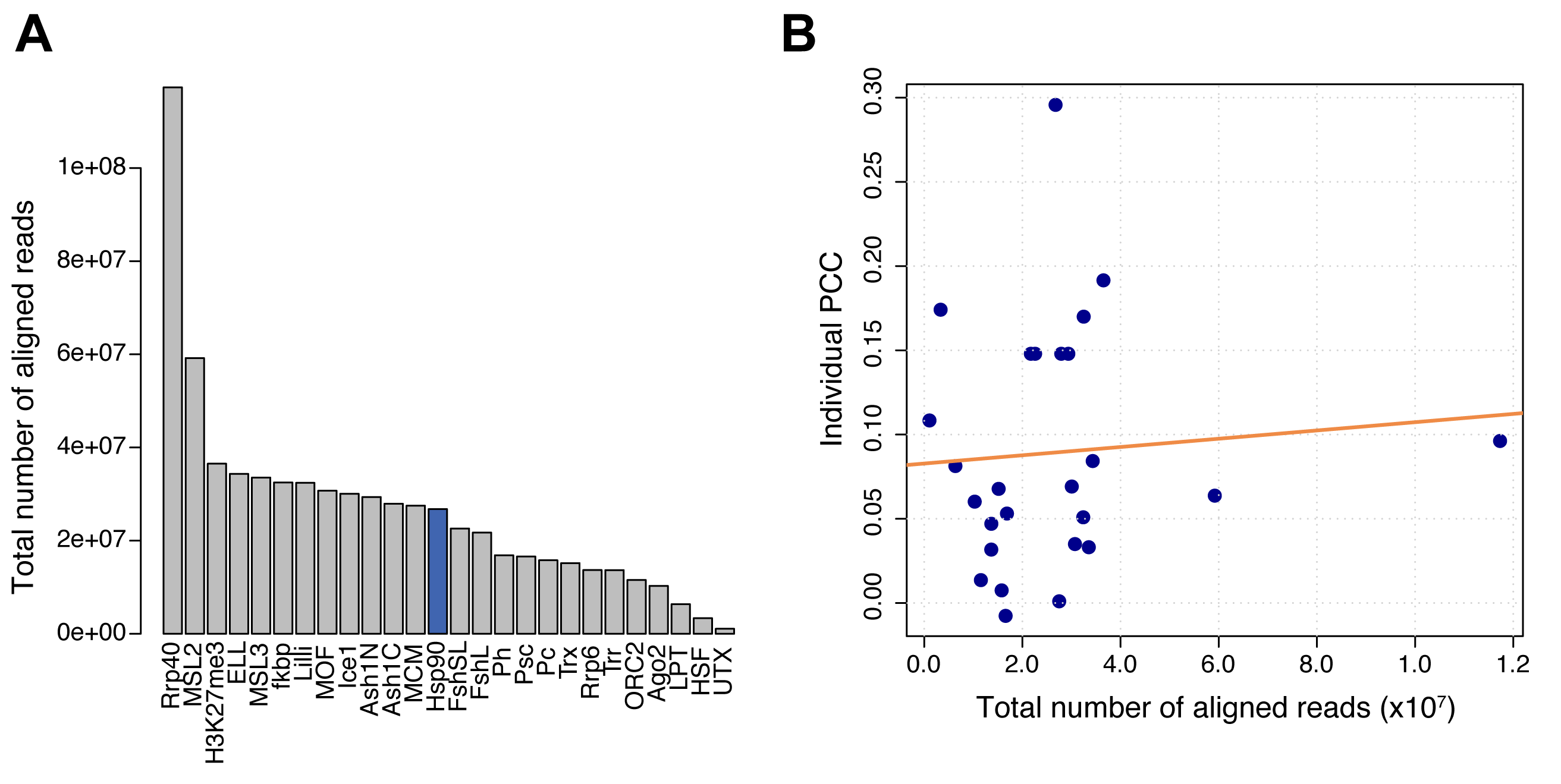

Supplement: Figure S5 — Sequencing depth analysis of ChIP-Seq-based chromatin features. (A) Sequencing depth, expressed as total number of aligned reads, for each ChIP-Seq profile included in this work. Features are ranked by decreasing coverage values and Hsp90 is highlighted in blue. (B) Individual predictive power of ChIP-Seq-based chromatin features as a function of their sequencing depth. Prediction accuracies are Pearson correlation coefficients. The orange lines indicates the fitted univariate linear regression model fit. (TIF) [file pcbi.1003419.s005.tif]

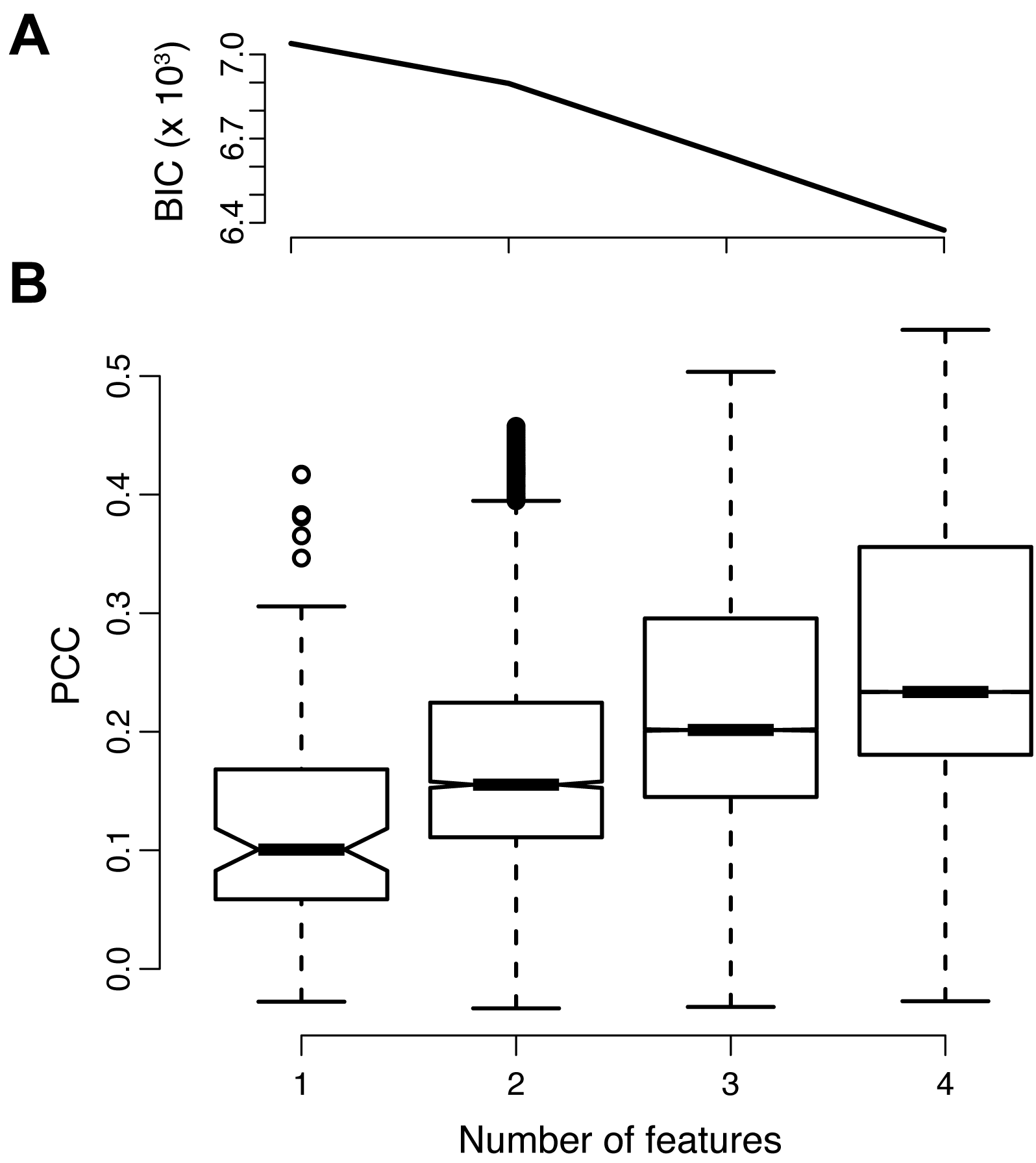

Supplement: Figure S6 — Prediction accuracies of simplified models obtained through exhaustive model search. (A) The value of the Bayesian Information Criterion (BIC) for the best simplified model (i.e. the model yielding the highest prediction accuracy (Pearson's correlation coefficient, PCC) in each group of one-, two-, three- and four-feature simplified models. (B) Boxplot of prediction accuracies for all simplified models within the same groups of models. (TIF) [file pcbi.1003419.s006.tif]

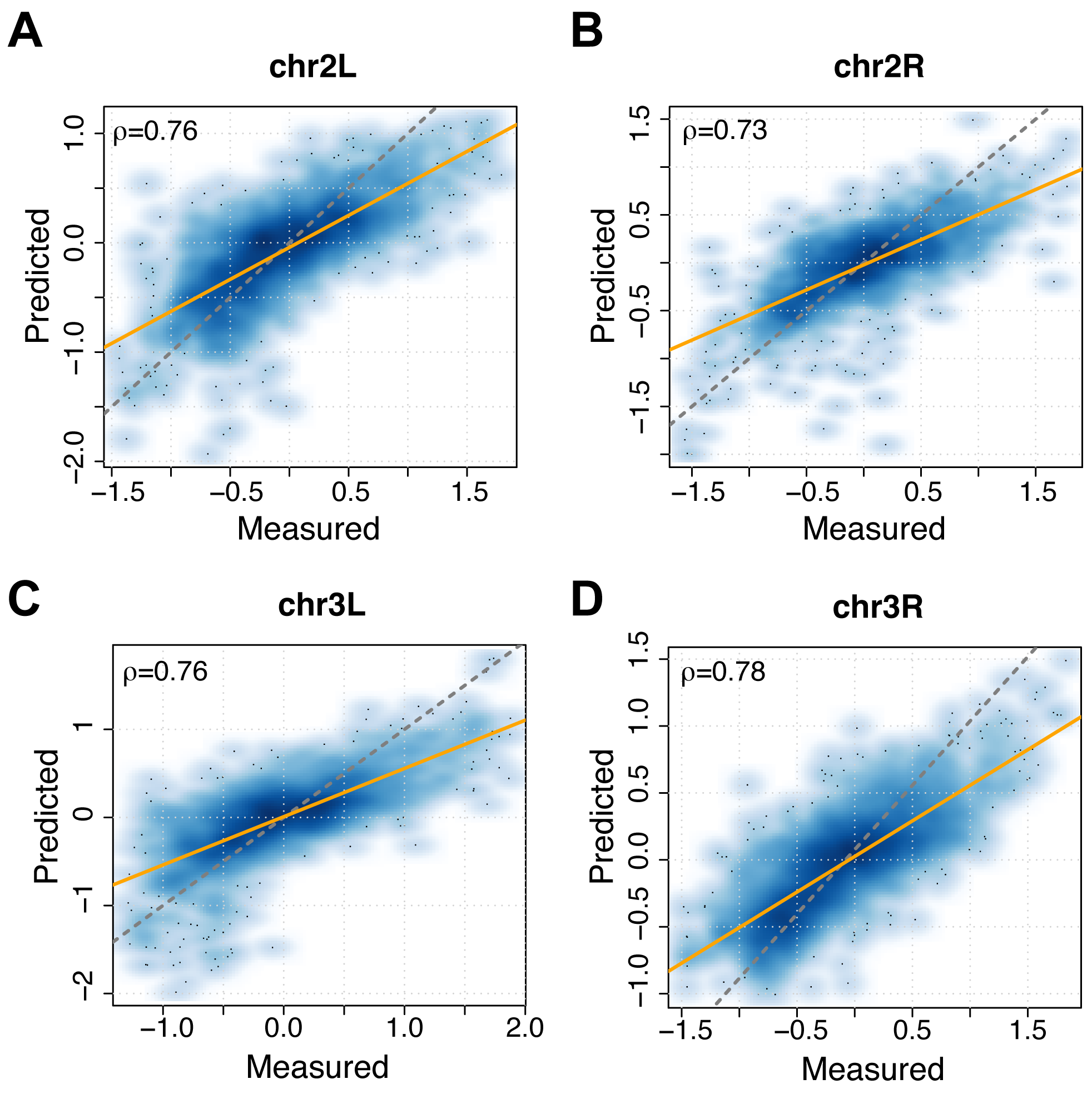

Supplement: Figure S7 — Predicting the replication timing of individual chromosome arms of the Drosophila S2 cells genome. Predicted versus experimentally measured replication timing of the Drosophila S2 cells genome for individual chromosome arms: (A) 2L (B) 2R (C) 3L (D) 3R. Model predictions were generated using chromatin binding proteins and second-order interactions between histone modifications (HMs2+CBPs) from a model trained at promoters. Prediction accuracies are Pearson correlation coefficients. Orange lines indicate the model fit, whereas dashed gray lines indicate the bisector . (TIF) [file pcbi.1003419.s007.tif]

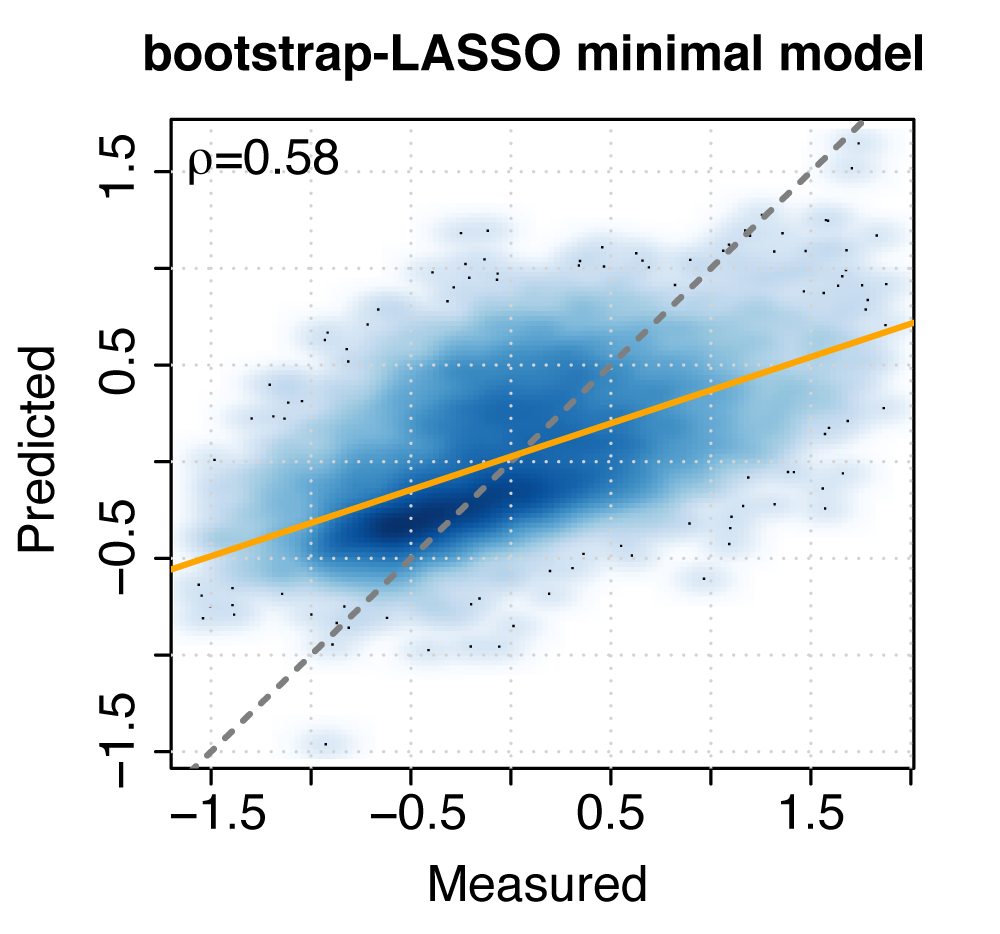

Supplement: Figure S8 — Prediction accuracy of the bootstrap-Lasso simplified model on the whole Drosophila S2 cells genome. Predicted versus experimentally measured replication timing of the Drosophila S2 cells genome represented as smoothed color density scatter plot. Model predictions were generated using the six-features (H4K8ac, H3K36me1, H2BUb, H2BUb:H3K36me1, H2BUb:H3K79me1 and Hsp90) bootstrap-Lasso simplified model trained at promoters. Prediction accuracy is Pearson correlation coefficient. The orange line indicates the model fit, whereas the dashed gray line indicates the bisector . (TIF) [file pcbi.1003419.s008.tif]

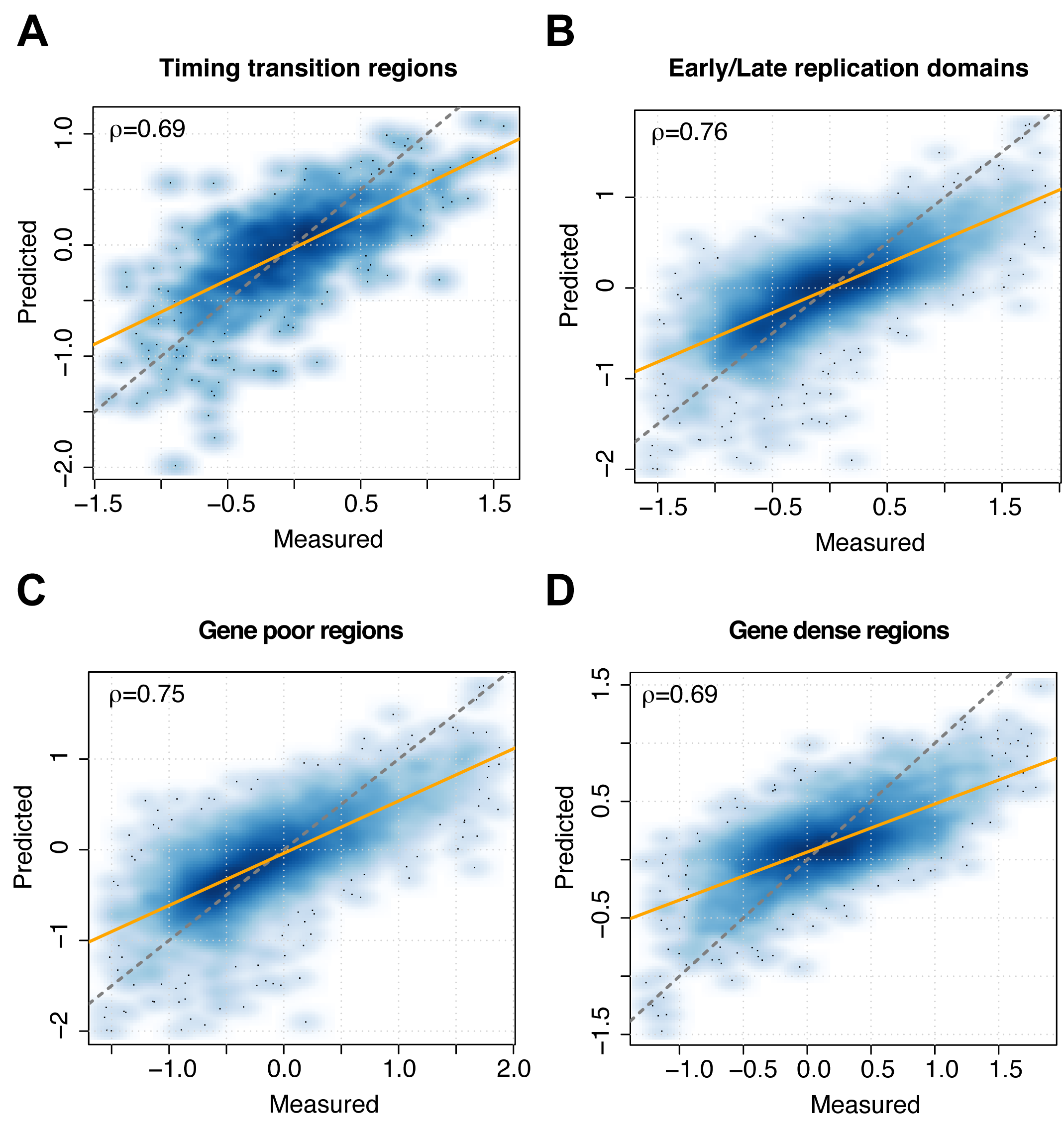

Supplement: Figure S9 — Evaluating prediction accuracies at different classes of genomic regions. Predicted versus experimentally measured replication timing of the Drosophila S2 cells genome at: (A) timing transition regions (B) early/late replication domains (C) gene poor regions (D) gene dense regions. Model predictions were generated using chromatin binding proteins and second-order interactions between histone modifications (HMs2+CBPs) from a model trained at promoters. Prediction accuracies are Pearson correlation coefficients. Orange lines indicate the model fit, whereas dashed gray lines indicate the bisector . (TIF) [file pcbi.1003419.s009.tif]

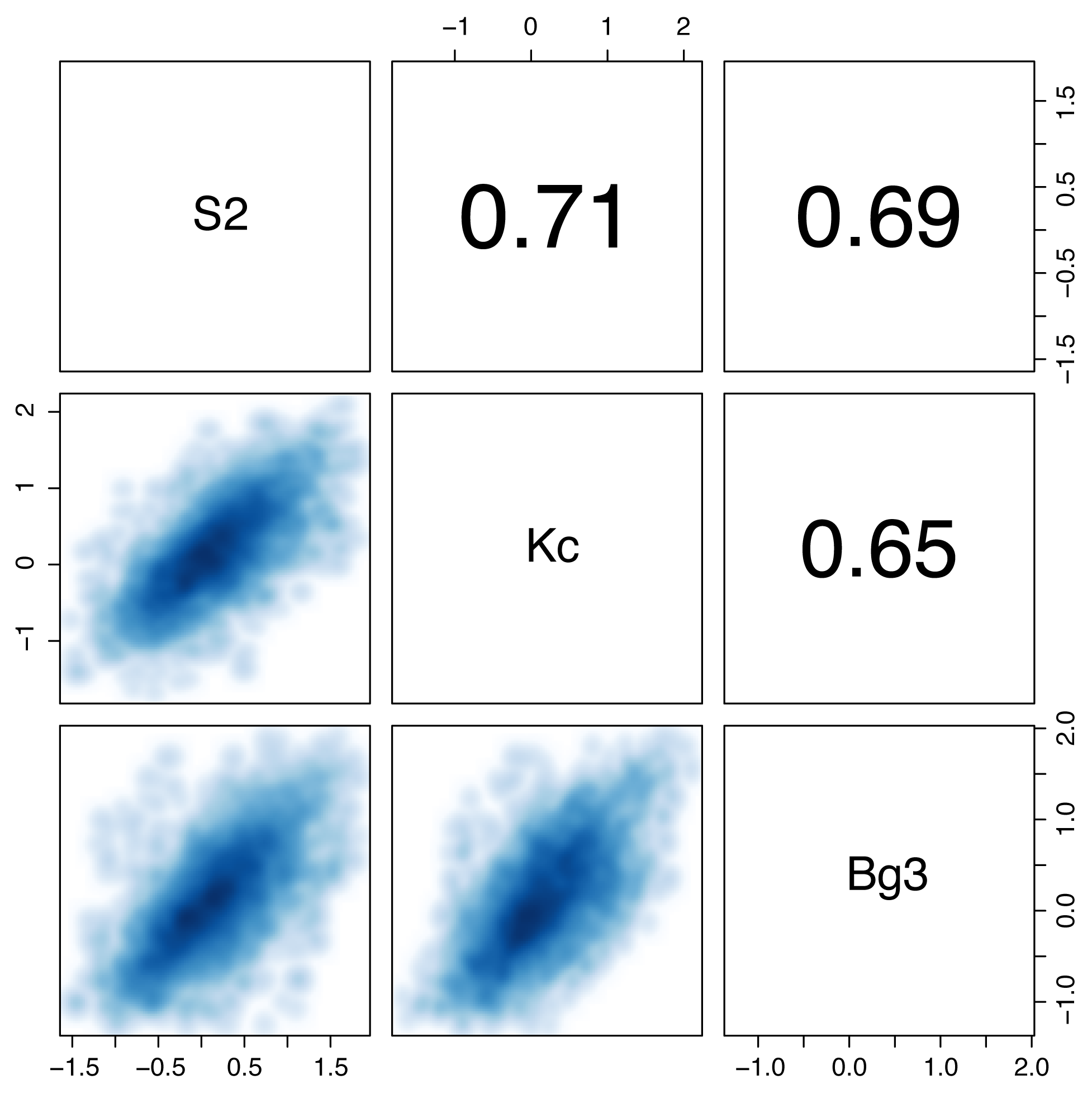

Supplement: Figure S10 — Correlation of DNA replication timing profiles at promoters in S2, Kc and Bg3 cell lines. Pairwise smoothed color density scatter plots between DNA replication timing of promoters in S2, Kc and Bg3 cell lines. Upper triangular entries are Pearson's correlation coefficients. (TIF) [file pcbi.1003419.s010.tif]

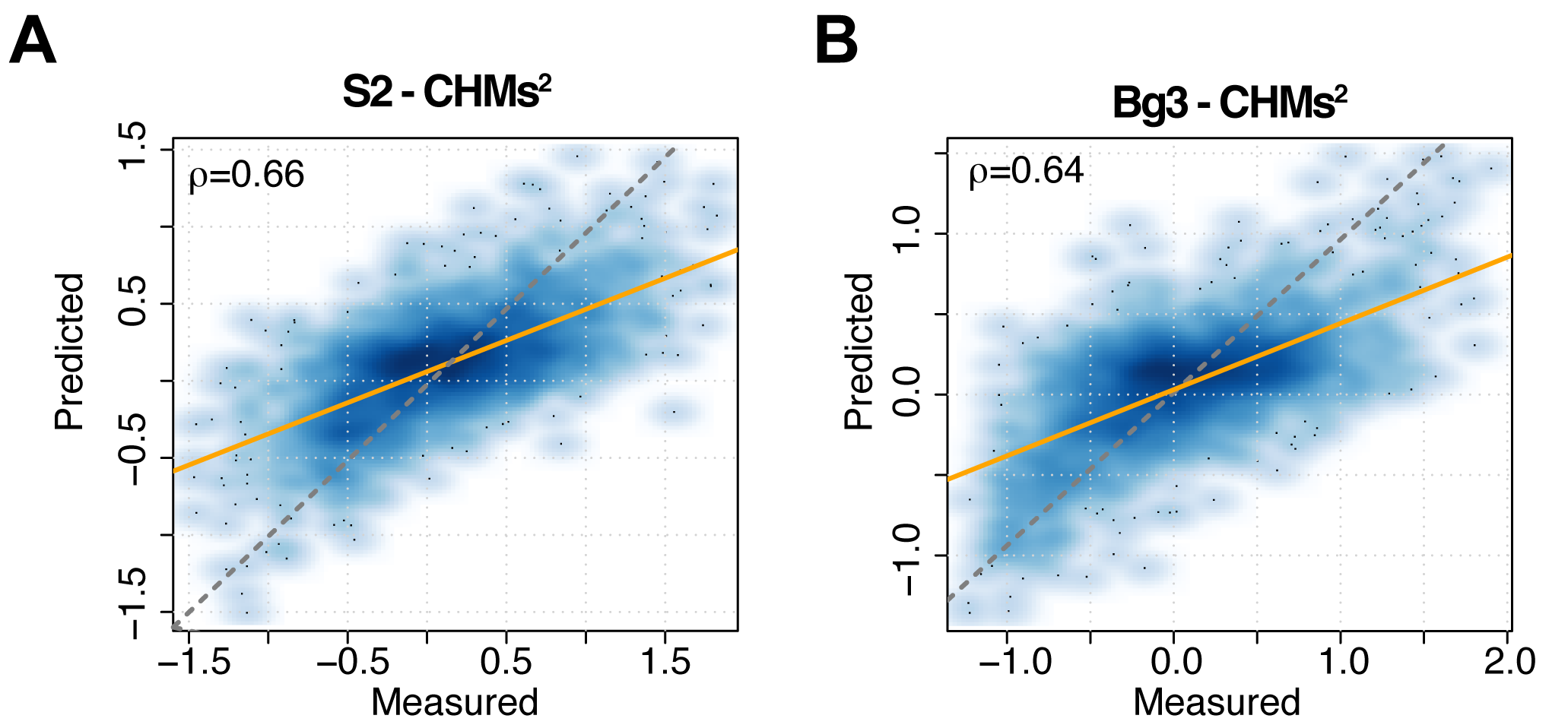

Supplement: Figure S11 — Evaluating the predictive power of HMs levels in common between S2 and Bg3 cells. Predicted versus experimentally measured replication timing of the test set represented as smoothed color density scatter plot. Model predictions were generated based on second-order interactions between HMs levels in S2 cells (A) and Bg3 cells (B), using only a subset of HMs that were profiled in both cell lines (CHMs2). Prediction accuracies are Pearson correlation coefficients. Orange lines indicate the model fit, whereas dashed gray lines indicate the bisector . (TIF) [file pcbi.1003419.s011.tif]

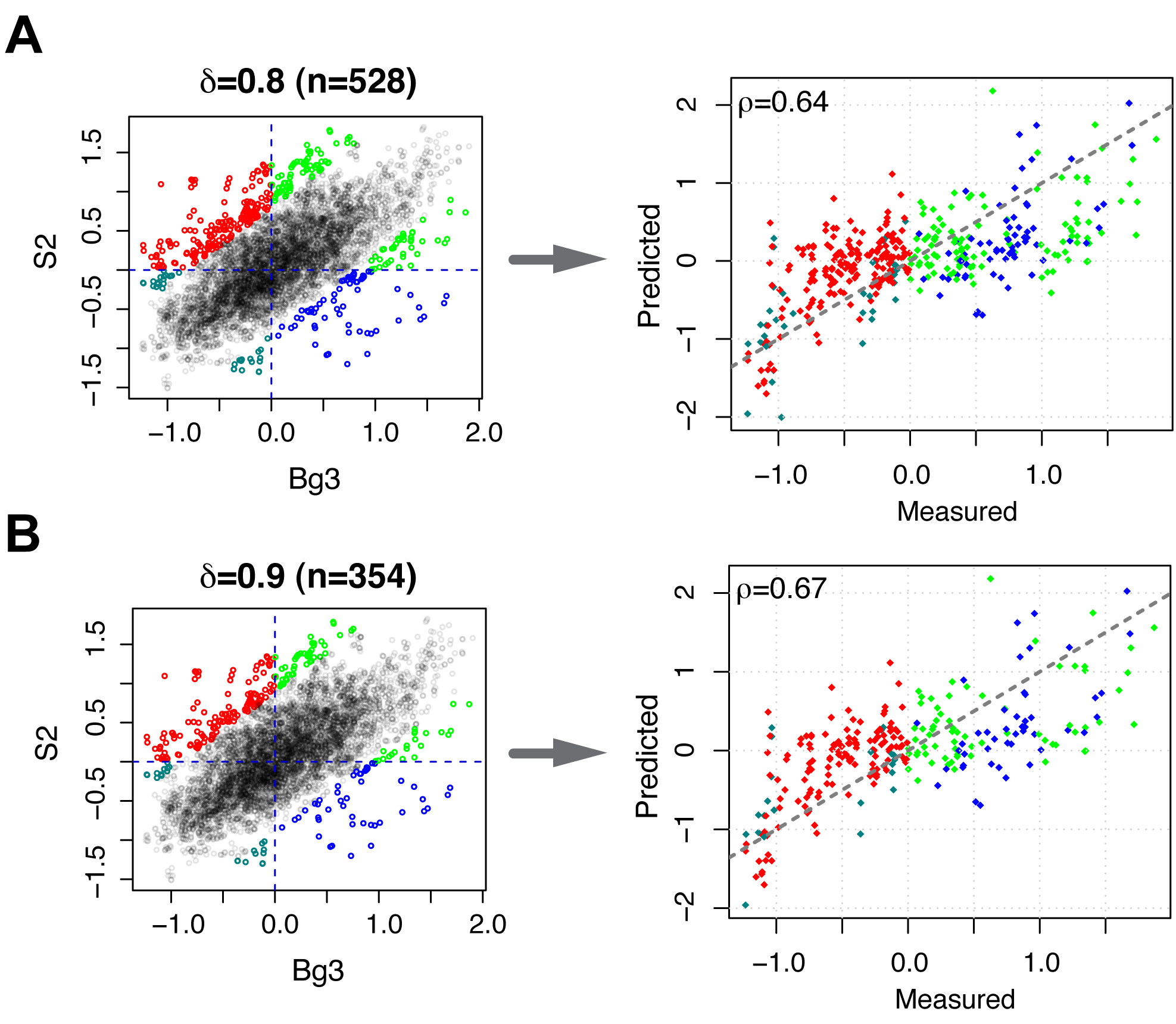

Supplement: Figure S12 — Predicting the replication timing of promoters that differentially replicate between S2 and Bg3 cells. (A, left) Replication timing of S2 cells promoters versus Bg3. Differentially replicating promoters are color-coded according to the quadrant (delimited by dashed blue lines) they belong to (red: early replicating in S2 and late replicating in Bg3; green: early in both S2 and Bg3; blue: late in S2 and early in Bg3, aqua: late in both S2 and Bg3). A total of n = 528 promoters exhibit a log fold change greater than or equal to 0.8 (δ = 0.8). (A, right) Experimentally determined replication timing in Bg3 versus predictions generated by a model based on pairwise interactions between CHMs in S2 cells. Prediction accuracy is Pearson correlation coefficient. The dashed gray line indicates the bisector . (B) Same as A, for δ = 0.9. (TIF) [file pcbi.1003419.s012.tif]
