# Supplementary material for: Combinatorial Modeling of Chromatin Features Quantitatively Predicts DNA Replication Timing in Drosophila
Source: PLoS Comput Biol. 2014 Jan 23;10(1):e1003419. doi: 10.1371/journal.pcbi.1003419 (PMC3900380; doi:10.1371/journal.pcbi.1003419)
Supplement: Table S1 — Pearson's correlation coefficients between measured and predicted replication timing for different sets of chromatin features and both Lasso and MARS statistical models. In addition, Lasso predictions on replication timing of S2 promoters are indicated for the model trained and tested using a second replication timing profile generated by Schwaiger et al. [37]. CBPs: chromatin binding proteins; HMs: histone modifications; HMs2+CBPs: CBPs and second-order multiplicative interactions between HMs; (HMs+CBPs)2: second-order multiplicative interaction terms of HMs and CBPs, encompassing pairwise interactions between HMs, CBPs and interactions between HMs and CBPs. (PDF) [file pcbi.1003419.s013.pdf]

Supporting Information for:

Combinatorial modeling of chromatin features quantitatively predicts DNA replication timing in *Drosophila*

Table S1

Federico Comoglio and Renato Paro

Chromatin features	Lasso	MARS	Lasso (Schwaiger et al.)
HMs^2	0.69	0.68	0.64
HMs^2+CBP	0.72	0.70	0.67
$(HMs + CBPs)^2$	0.74	0.73	0.69

References

[1] Schwaiger,M., Stadler,M.B., Bell,O., Kohler,H., Oakeley,E.J. and Schubeler,D. (2009) Chromatin state marks cell-type- and gender-specific replication of the Drosophila genome. *Genes Dev.*, **23**, 589-601.
