# Supplementary material for: Combinatorial Modeling of Chromatin Features Quantitatively Predicts DNA Replication Timing in Drosophila
Source: PLoS Comput Biol. 2014 Jan 23;10(1):e1003419. doi: 10.1371/journal.pcbi.1003419 (PMC3900380; doi:10.1371/journal.pcbi.1003419)
Supplement: Table S2 — Summary of the chromatin binding protein profiles included in the analysis. This table provides details and accession numbers of the chromatin binding protein profiles used for modeling. (PDF) [file pcbi.1003419.s014.pdf]

Supporting Information for:

Combinatorial modeling of chromatin features quantitatively predicts DNA replication timing in *Drosophila*

Table S2

Federico Comoglio and Renato Paro

Flybase name	Symbol	Source	Identifier
Argonaute 2	AGO2	SRA	SRP002788
absent, small, or homeotic discs, C-ter domain 1	Ash1C	GEO	GSE30820 [1]
absent, small, or homeotic discs, N-ter domain 1	Ash1N	GEO	GSE30820 [1]
Boundary element-associated factor of 32 kDa	BEAF	modENCODE	modENCODE_274
blanks	CG10630	modENCODE	modENCODE_4176
Chromator	Chro	modENCODE	modENCODE_278
Centrosomal protein of 190 kDa	CP190	modENCODE	modENCODE_925
CCCTC-binding factor	CTCF	modENCODE	modENCODE_283
dMi-2	dMi-2	modENCODE	modENCODE_3676
Sex combs extra	dRING	modENCODE	modENCODE_928
Scm-related gene containing four mbt domains	dSFMBT	modENCODE	modENCODE_3751
Utx	dUTX	SRA	SRR585048 [5]
Su(Tpl)	ELL	SRA	SRR345566 [6]
Enhancer of Zeste	E(z)	modENCODE	modENCODE_2988
female sterile (1) homeotic L	FshL	GEO	GSE30820 [1]
female sterile (1) homeotic SL	FshSL	GEO	GSE30820 [1]
FK506-binding protein 2	fkbp	in-house	†
Suppressor of variegation 205	HP1a	modENCODE	modENCODE_2668
Heterochromatin Protein 1b	HP1b	modENCODE	modENCODE_941
Heterochromatin Protein 1c	HP1c	modENCODE	modENCODE_943
rhino	HP1d	modENCODE	modENCODE_4187
Heterochromatin Protein 2	HP2	modENCODE	modENCODE_944
Heterochromatin Protein 4	HP4	modENCODE	modENCODE_3948
Heat shock factor	HSF	SRA	SRX018631 [7]
Heat shock protein 83	Hsp90	GEO	GSE31226 [2]
Imitation SWI	ISWI	modENCODE	modENCODE_3032
Interacts with the C terminus of ELL 1	Ice1	SRA	SRR345567 [6]
Suppressor of variegation 3-1	JIL-1	modENCODE	modENCODE_945
Lysine (K)-specific demethylase 2	Kdm2	modENCODE	modENCODE_3033
Histone demethylase 4A	Kdm4A	modENCODE	modENCODE_3784
lilliputian	Lilli	SRA	SRR345568 [6]
lost PHD finger of Trr	LPT	SRA	SRR585049 [5]
MBD-R2	MBD-R2	modENCODE	modENCODE_946
Minichromosome maintenance complex component 2-7	MCM	SRA	SRP006146
maleless	MLE	modENCODE	modENCODE_3788
modifier of mdg4	MOD2.2	modENCODE	modENCODE_2674
males absent on the first	MOF	SRA	SRR495372, SRR495373 [8]
MRG15	MRG15	modENCODE	modENCODE_3047
male-specific lethal 1	MSL1	modENCODE	modENCODE_3293
male-specific lethal 2	MSL2	SRA	SRR495368, SRR495369 [8]
male-specific lethal 3	MSL3	SRA	SRR495370, SRR495371 [8]
Enhancer of bithorax	NURF	modENCODE	modENCODE_947

Continued on next page

Table S2 – continued from previous page

Flybase name	Symbol	Source	Identifier
Origin Recognition Complex subunit 2	ORC2	SRA	SRP002091
Polycomb	Pc	GEO	GSE24521 [3]
Polycomblike	PCL	modENCODE	modENCODE_3049
Polyhomeotic	Ph	GEO	GSE24521 [3]
pleiohomeotic	Pho	modENCODE	modENCODE_3894
Painting of fourth	POF	modENCODE	modENCODE_3294
RNA polymerase II 215kD subunit II	PolIICC*	modENCODE	modENCODE_329
Rrp6	Rrp6	SRA	SRR610262, SRR610263 [9]
Rrp40	Rrp40	SRA	SRR610264, SRR610265 [9]
PR-Set7	PR-Set7	modENCODE	modENCODE_3054
Posterior sex combs	Psc	GEO	GSE24521 [3]
RPD3	RPD3	modENCODE	modENCODE_3057
dre4	SPT16	modENCODE	modENCODE_3058
Suppressor of Hairy wing	Su(Hw)	modENCODE	modENCODE_330
Suppressor of variegation 3-3	Su(var)3-3	modENCODE	modENCODE_3949
Suppressor of variegation 3-7	Su(var)3-7	modENCODE	modENCODE_2672
Suppressor of variegation 3-9	Su(var)3-9	modENCODE	modENCODE_2673
Topoisomerase II	TopoII	modENCODE	modENCODE_3945
Trithorax-like	Trl	modENCODE	modENCODE_285
Trithorax-related	Trr	SRA	SRR585047 [5]
Trithorax	Trx	GEO	GSE24521 [3]
will die slowly	WDS	modENCODE	modENCODE_953
deformed wings	ZW5	modENCODE	modENCODE_3804

†: unpublished data (manuscript in preparation).

References

[1] Kockmann,T., Gerstung,M., Schlumpf,T., Xhinzhou,Z., Hess,D., Beerenwinkel,N., Beisel,C. and Paro,R. (2013) The BET protein FSH functionally interacts with ASH1 to orchestrate global gene activity in Drosophila. *Genome Biol.*, in press

[2] Sawarkar,R., Sievers,C. and Paro,R. (2012) Hsp90 globally targets paused RNA polymerase to regulate gene expression in response to environmental stimuli. *Cell*, **149**, 807-818.

[3] Enderle,D., Beisel,C., Stadler,M.B., Gerstung,M. Athri,P. and Paro,R. (2011) Polycomb preferentially targets stalled promoters of coding and noncoding transcripts. *Genome Res.*, **21**, 216-226.

[4] Schwaiger,M., Stadler,M.B., Bell,O., Kohler,H., Oakeley,E.J. and Schubeler,D. (2009) Chromatin state marks cell-type- and gender-specific replication of the Drosophila genome. *Genes Dev.*, **23**, 589-601.

[5] Herz HM, Mohan M, Garruss AS, Liang K, Takahashi YH et al (2012) Enhancer-associated H3K4 monomethylation by Trithorax-related, the Drosophila homolog of mammalian Mll3/Mll4. Genes Dev 26(23): 2604-20.

[6] Smith ER, Lin C, Garrett AS, Thornton J, Mohaghegh N et al (2011) The little elongation complex regulates small nuclear RNA transcription. Mol Cell 44(6): 954-965.

[7] Guertin MJ, Lis JT (2010) Chromatin landscape dictates HSF binding to target DNA elements. PLoS Genet 6(9): e1001114.

[8] Straub T, Zabel A, Gilfillan GD, Feller C, Becker PB (2013) Different chromatin interfaces of the Drosophila dosage compensation complex revealed by high-shear ChIP-seq. Genome Res. 23(3): 473-85.

[9] Lim SJ, Boyle PJ, Chinen M, Dale RK, Lei EP (2013) Genome-wide localization of exosome components to active promoters and chromatin insulators in Drosophila. Nucleic Acids Res 41(5): 2963-2980.
