# Supplementary material for: Combinatorial Modeling of Chromatin Features Quantitatively Predicts DNA Replication Timing in Drosophila
Source: PLoS Comput Biol. 2014 Jan 23;10(1):e1003419. doi: 10.1371/journal.pcbi.1003419 (PMC3900380; doi:10.1371/journal.pcbi.1003419)
Supplement: Table S3 — Summary of the histone modification profiles included in the analysis. This table provides details and accession numbers of the histone modification profiles used for modeling. (PDF) [file pcbi.1003419.s015.pdf]

Supporting Information for:

Combinatorial modeling of chromatin features quantitatively predicts DNA replication timing in *Drosophila*

Table S3

Federico Comoglio and Renato Paro

Symbol	Source	Identifier
H1	modENCODE	modENCODE_3300
H2Av	modENCODE	modENCODE_2991
H2BK5ac	modENCODE	modENCODE_3283
H2BUb	modENCODE	modENCODE_290
H3	modENCODE	modENCODE_3301
H3.3	modENCODE	modENCODE_2528
H3K4me1	modENCODE	modENCODE_304
H3K4me2	modENCODE	modENCODE_2655
H3K4me3	modENCODE	modENCODE_305
H3K9ac	modENCODE	modENCODE_309
H3K9acS10P	modENCODE	modENCODE_2660
H3K9me1	modENCODE	modENCODE_3770
H3K9me2	modENCODE	modENCODE_3011
H3K9me3	modENCODE	modENCODE_313
H3K18ac	modENCODE	modENCODE_292
H3K23ac	modENCODE	modENCODE_294
H3K27ac	modENCODE	modENCODE_296
H3K27me1	modENCODE	modENCODE_3943
H3K27me2	modENCODE	modENCODE_3000
H3K27me3	SRA	SRR585053 [1]
H3K36me1	modENCODE	modENCODE_3170
H3K36me3	modENCODE	modENCODE_303
H3K79me1	modENCODE	modENCODE_2658
H3K79me2	modENCODE	modENCODE_307
H4	modENCODE	modENCODE_3304
H4acTetra	modENCODE	modENCODE_201
H4K5ac	modENCODE	modENCODE_321
H4K8ac	modENCODE	modENCODE_322
H4K16ac	modENCODE	modENCODE_320
H4K20me	modENCODE	modENCODE_3014

References

[1] Herz H-M, Mohan M, Garruss AS, Liang K, Takahashi Y et al. (2012) Enhancer-associated H3K4 monomethylation by Trithorax-related, the Drosophila homolog of mammalian Mll3/Mll4. Genes Dev 26: 2604-2620.
